# Structural mechanism for signal transduction in RXR nuclear receptor heterodimers

**DOI:** 10.1038/ncomms9013

**Published:** 2015-08-20

**Authors:** Douglas J. Kojetin, Edna Matta-Camacho, Travis S. Hughes, Sathish Srinivasan, Jerome C. Nwachukwu, Valerie Cavett, Jason Nowak, Michael J. Chalmers, David P. Marciano, Theodore M. Kamenecka, Andrew I. Shulman, Mark Rance, Patrick R. Griffin, John B. Bruning, Kendall W. Nettles

**Affiliations:** 1 Department of Molecular Therapeutics, The Scripps Research Institute-Scripps Florida, 130 Scripps Way, Jupiter, Florida 33458, USA; 2 Department of Cancer Biology, The Scripps Research Institute-Scripps Florida, 130 Scripps Way, Jupiter, Florida 33458, USA; 3 Department of Pharmacology, University of Texas Southwestern Medical Center, Dallas, Texas 75390, USA; 4 Department of Molecular Genetics, Biochemistry and Microbiology, University of Cincinnati, Cincinnati, Ohio 45267, USA; 5 School of Biological Sciences, The University of Adelaide, Adelaide, South Australia 5005, Australia

## Abstract

A subset of nuclear receptors (NRs) function as obligate heterodimers with retinoid X receptor (RXR), allowing integration of ligand-dependent signals across the dimer interface via an unknown structural mechanism. Using nuclear magnetic resonance (NMR) spectroscopy, x-ray crystallography and hydrogen/deuterium exchange (HDX) mass spectrometry, here we show an allosteric mechanism through which RXR co-operates with a permissive dimer partner, peroxisome proliferator-activated receptor (PPAR)-γ, while rendered generally unresponsive by a non-permissive dimer partner, thyroid hormone (TR) receptor. Amino acid residues that mediate this allosteric mechanism comprise an evolutionarily conserved network discovered by statistical coupling analysis (SCA). This SCA network acts as a signalling rheostat to integrate signals between dimer partners, ligands and coregulator-binding sites, thereby affecting signal transmission in RXR heterodimers. These findings define rules guiding how NRs integrate two ligand-dependent signalling pathways into RXR heterodimer-specific responses.

## Abstract (web summary)

Some nuclear receptors dimerize with retinoid X receptor to allow ligand-dependent signalling. Here, Kojetin *et al.* use structural and biophysical techniques to identify structural changes that guide these complex signalling networks.

## Graphical abstract

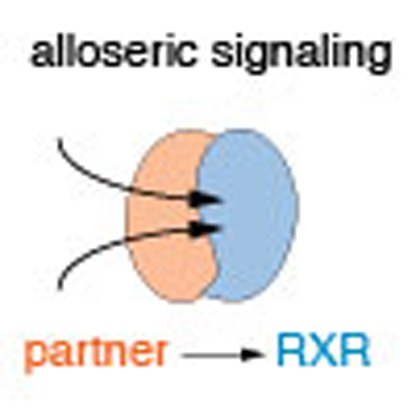

The nuclear receptor (NR) superfamily of transcription factors are broadly implicated in metazoan physiology, and modulate gene expression in response to steroids, lipids, bile acids and other small lipophilic molecules or synthetic ligands^1^. NRs harbour a C-terminal ligand-binding and transactivation domain (LBD), a central DNA-binding domain and a variable N-terminal disordered transactivation domain. These receptors transduce signals from ligand binding in the LBD to regulate gene expression by recruiting co-regulator proteins that modify chromatin and the associated transcriptional complex^2^.

The physical mechanisms governing allosteric signalling between NR ligands and coregulator-binding sites remain poorly understood. Allosteric control of NR function is modulated by a number of factors, including cell type-specific co-regulators^3^, post-translational modifications^4^,^5^, DNA recognition elements^6^,^7^,^8^ and NR heterodimer partners^9^,^10^,^11^. Understanding the complex allosteric signalling of NRs requires first dissecting the signalling mechanisms within individual domains and binding sites, which will facilitate understanding the more difficult questions related to inter-domain communication^12^. Structural studies have revealed mechanisms that direct communication between ligand and coregulator-binding sites within a single LBD^13^,^14^. The fully active LBD conformer is well-characterized^15^,^16^,^17^ and its conformation is conserved within the context of the full-length receptor^18^. In its agonist-stabilized conformation, the C-terminal helix, helix 12 forms one side, while helices 3–5 form the other sides of a co-regulator-binding site called the Activation Function-2 (AF-2) surface. Some NR antagonists, such as tamoxifen or RU486, contain a pendent side group that physically relocates helix 12 out of the active conformation thus blocking co-activator recruitment^15^,^19^,^20^. More recently, we identified a fine-tuning mechanism for indirectly modulating helix 12 conformation, allowing NRs to direct a graded range of signalling outputs from partial to full agonist^21^,^22^,^23^,^24^. We have also defined a structural mechanism whereby graded agonists and non-agonists do not fully stabilize the conformational dynamics of the AF-2 surface^4^,^25^,^26^,^27^. However, it is poorly understood how ligand binding to one LBD controls co-regulator recruitment to its dimer partner within a NR heterodimer complex.

A subset of NRs functions as heterodimers with retinoid X receptor (RXR), and thus provides a mechanism to integrate two distinct ligand signalling pathways^28^. In some contexts, RXR heterodimers can act as two independent signalling moieties^29^. However, allosteric phenomena between RXR and partner are not well-understood. First, some heterodimer partners, such as the peroxisome proliferator-activated receptor-γ (PPARγ), farnesoid X receptor and liver X receptor (LXR), are ‘permissive’ for RXR activity, where the heterodimer is strongly activated by ligands for either partner in the dimer^30^,^31^. However, the integration of signals varies with both receptor and ligand combinations, which can produce either additive or synergistic effects^32^,^33^. Second, RXR heterodimers that contain retinoic acid receptor (RAR), vitamin D receptor (VDR) or thyroid hormone receptor (TR), are ‘non-permissive’ for RXR as they generally do not respond to RXR ligands^34^, or do so only in certain contexts in the presence of the partner ligand^35^,^36^. The structural mechanisms that generate this spectrum of signalling outcomes are unknown.

Here we present comprehensive structural analyses of a ‘permissive’ (PPARγ/RXRα) and ‘non-permissive’ (TR/RXR) heterodimeric complex, which defines how a non-permissive dimer partner allosterically silences RXR. Solution nuclear magnetic resonance (NMR) spectroscopy reveals a mechanism by which the liganded state of TR, but not PPARγ, uniquely affects the conformational dynamics of RXR. A crystal structure of the TR/RXR heterodimer defines a structural mechanism for this silencing, which occurs through a sequence of conformational relays between the helix 11 pairs that constitute most of the dimer interface, transferred to a rotation of helix 5 in the core of the RXR LBD, leading to disruption of the adjacent co-regulator- and ligand-binding sites. This allosteric signalling pathway is further confirmed by NMR and hydrogen/deuterium exchange (HDX) mass spectrometry. Notably, analysis of other NR dimers reveals that these structural changes are part of an evolutionarily conserved energetic network, defined by a statistical coupling analysis (SCA) method^10^, where helix 5 functions more generally as a signalling rheostat that integrates signals with the dimer interface, ligand and coregulator-binding sites.

## Results

### Conformational dynamics control RXR permissiveness

**Figure 1:**
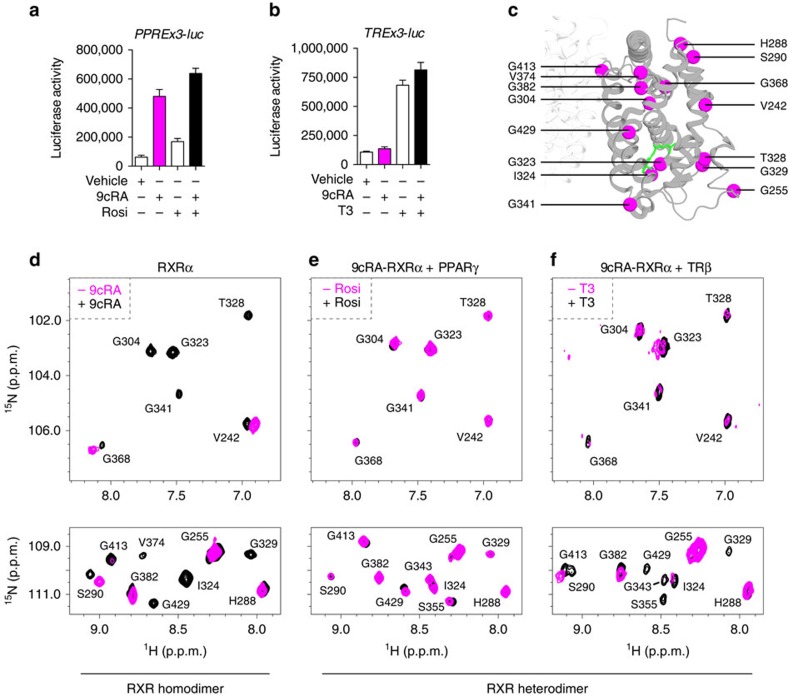
NMR reveals a role for conformational dynamics in RXR permissiveness. (**a**) CV-1 cells co-transfected with PPARγ expression plasmid, RXRα expression plasmid and a PPAR-responsive luciferase reporter. Cells were treated with vehicle, 10 μM 9cRA and/or 10 μM rosiglitazone for 24 h. Magenta and black bars are coloured to match NMR data in **d**, where ligand content is the same. Luciferase activity is shown normalized to vehicle-treated cells and was performed in quadruplicate, plotted with the average (±s.e.m) and representative of at least three experiments. (**b**) CV-1 cells co-transfected with TRβ expression plasmid, RXRα expression plasmid and a TR-responsive luciferase reporter. Cells were treated with vehicle, 100 nM LG100268 (LG268) and/or 1 μM T3 for 24 h. Luciferase activity is shown normalized to vehicle-treated cells and was performed in quadruplicate, plotted with the average (±s.e.m) and representative of at least two experiments. Magenta and black bars are coloured to match NMR data in **e**, where ligand content is the same. (**c**) Structural location of RXR residues mentioned in the NMR analysis. (**d**) Overlay of 2D [^1^H,^15^N]-TROSY-HSQC NMR data for [^2^H,^13^C,^15^N]-RXRα LBD in the apo form and coloured magenta—with the same bound to 9cRA and coloured black. (**e**) Overlay of 2D [^1^H,^15^N]-TROSY-HSQC NMR data for [^2^H,^13^C,^15^N]-RXRα LBD bound to 9cRA and heterodimerized to apo-PPARγ and coloured magenta—with the same bound to rosiglitazone (Rosi) and coloured black. (**f**) Overlay of 2D [^1^H,^15^N]-TROSY-HSQC NMR data for [^2^H,^13^C,^15^N]-RXRα LBD bound to 9cRA and heterodimerized to apo-TRβ and coloured magenta—with the same bound to T3 and coloured black.

The RXR agonist, 9-*cis*-retinoic acid (9cRA), stimulates transactivation of PPARγ/RXRα (Fig. 1a), verifying PPARγ as a permissive RXRα partner. However, 9cRA and another RXR agonist, LG100268 (LG268), have no effect on TRβ/RXRα (Fig. 1b) and VDR/RXRα (Supplementary Fig. 1), establishing TRβ and VDR as non-permissive RXR partners. To gain insight into the structural basis for this heterodimer-specific signalling, we performed solution NMR on isotopically labelled RXRα LBD alone as a homodimer, and in complex with unlabelled PPARγ LBD or TRβ LBD as heterodimers. This analysis enabled us to specifically observe conformational effects in RXRα that result from ligand binding to its heterodimer partner.

NMR resonances corresponding to residues within the apo-RXRα ligand-binding pocket and AF-2 surface are missing or have broad linewidths. These regions exist as a dynamic ensemble of conformations, exchanging between two or more conformations in a molten globule-like state on the μs–ms timescale^37^,^38^,^39^. Binding of 9cRA to the RXRα homodimer stabilizes the ligand-binding pocket and AF-2 surface^37^ (Fig. 1b,c), resulting in the appearance and sharpening of NMR resonances relative to apo-RXRα. Thus, the NMR-observed structural mechanism by which an agonist activates RXRα occurs by stabilizing an active conformation. That is, agonist binding quenches the μs–ms conformational dynamics of the apo-RXRα ligand-binding pocket and surrounding regions, including the AF-2 surface. This mechanism is also supported by HDX mass spectrometry studies, which demonstrate stabilization of the RXRα ligand-binding pocket and AF-2 surface on ligand binding^40^,^41^,^42^. This conformational activation phenotype, whereby the dynamics of the apo-NR LBD is affected (stabilized) by agonist binding, has been observed for PPARγ^25^,^26^,^39^, VDR^43^,^44^, constitutive androstane receptorc^45^ and other receptors, indicating this may be a general feature for ligand activation of NRs.

To determine the mechanism through which PPARγ acts as a permissive dimer partner, we performed differential NMR analysis by adding unlabelled apo-PPARγ to 9cRA-bound isotopically labelled RXRα, with and without addition of the full PPARγ agonist, rosiglitazone (Fig. 1b,d). NMR chemical shift changes in RXRα are observed on addition of apo-PPARγ to the 9cRA-bound RXRα, consistent with complete formation of a heterodimer complex. Addition of rosiglitazone causes subtle but significant NMR chemical shift changes in RXRα (for example, S355 and G429 in the dimer interface) and only minor changes in NMR resonance linewidths for select residues. Thus, although heterodimerization with and ligand binding to PPARγ perturbs the conformation of RXRα, neither of these events dramatically affects the μs–ms dynamics of RXRα. This is in contrast to what occurs with the non-permissive RXR partner, TRβ, as detailed below.

To determine the mechanism through which TRβ acts as a non-permissive RXR heterodimer partner, we performed differential NMR analysis by adding unlabelled apo-TRβ to 9cRA-bound isotopically labelled RXRα, with and without addition of the TRβ agonist, T3 (Fig. 1b,e). In contrast to PPARγ, addition of apo-TRβ exerts a profound effect on the μs–ms conformational dynamics of 9cRA-bound RXRα, where a large number of agonist-bound RXRα NMR resonances revert to an apo-like NMR profile. NMR resonances that are destabilized—missing or have broad linewidths indicating increased μs–ms motion—correspond to RXRα residues in the ligand-binding pocket (for example, I324, G323, T328, G329, G341, G343 and S355), helix 11 (for example, G413 and G429) and other nearby regions such as helix 8 (for example, G368). Even more striking is that the addition of the TRβ agonist, T3, re-stabilizes these agonist-bound RXRα residues by decreasing motion on the μs–ms timescale, resulting in a reappearance of NMR resonances for these regions. Notably, many of the missing NMR resonances in the apo-TRβ/agonist-RXRα heterodimer correspond to residues in the apo-RXRα homodimer that are stabilized on binding 9cRA. Our NMR studies indicate that these residues are not affected by PPARγ heterodimerization or ligand binding to PPARγ, but they are significantly affected by TRβ heterodimerization and ligand binding to TRβ. In total, these data indicate that the mechanism through which RXRα is allosterically silenced by TRβ but not PPARγ, involves conformational dynamics on the μs–ms timescale.

### Structure of the TRβ·T3/apo-RXRα complex

**Figure 2:**
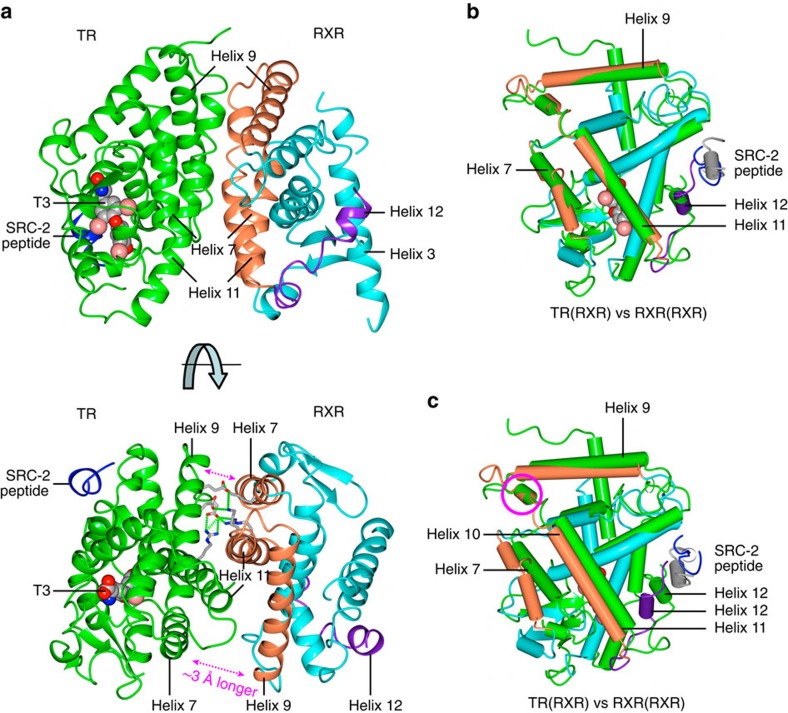
Crystal structure of the TRβ·T3·SRC-2/apo-RXRα LBD complex. (**a**) Structure of the TR/RXR LBD heterodimer is shown as ribbon with T3 as space filled. TR is coloured green, and the bound SRC-2 peptide is coloured blue and only binds to T3·TR. RXR is coloured light blue, with the dimer interface coloured coral and helix 12 coloured purple. There is no bound ligand in RXR, and RXR helix 12 adopts an inactive conformation positioned into the AF-2 co-activator-binding surface. (**b**) TR is superimposed on the RXR LBD homodimer (PDB 1MVC), showing conservation of domain structure. Structures are coloured as in **a**. (**c**) Same as **b**, except dimers are superimposed via the RXR protomer, rather than TR to RXR, illustrating the shift in the TR dimer interface relative to the other RXR promoter in the RXR homodimer. The magenta circle highlights the only region that superimposes similarly between TR and RXR, the amino-terminal end of TRβ helix 11.

To further detail the structural mechanism by which TRβ allosterically silences RXRα, we crystallized apo-RXRα in complex with TRβ, T3 and a co-activator peptide derived from SRC-2. The anisotropic data set was scaled to a resolution of 3.2–3.8 Å and refined to an *R*_work_/*R*_free_ of 23.6/28.1% (Table 1). Consistent with other RXR heterodimer structures, TRβ and RXRα interact via the conserved dimer interface, largely comprised of helix 11 in each monomer, with additional contacts from helices 8 and 10 (Fig. 2a). TRβ adopts the active conformation when bound to T3, with helix 12 forming one side of the co-activator-binding site, allowing the docking of the SRC-2 peptide. RXRα displays an inactive conformation with no bound ligand or co-activator peptide while helix 12 docks at the AF-2 surface. Although the asymmetric unit of the TRβ·T3/apo-RXRα complex is a dimer, the crystal packing reveals a heterotetramer assembly (Supplementary Fig. 2).

Compared with other NR heterodimer structures, TRβ·T3/apo-RXRα displays a more extreme deviation from the C2 (180°) symmetry of the dimer. RXRα helix 7 forms an extensive hydrogen bond network with TRβ helix 9 (Fig. 2a). However, the symmetry-related RXRα helix 9 and TRβ helix 7 are further apart by ∼3 Å, preventing this sort of interaction. Superposing TRβ with an RXRα subunit in the homodimer structure clearly revealed that the overall LBD structure is highly conserved (Fig. 2b). However, superposing these two structures via the RXRα protomer of the dimer subunits revealed a dramatic shift in the dimer interface (Fig. 2c). The amino-terminal end of TRβ helix 11 is oriented similarly towards RXRα, while TRβ helices 7, 10 and the carboxyl-terminal part of helix 11 are substantially shifted. As discussed below, this altered dimer interface induces conformational changes in RXRα, accounting for its silencing by TRβ.

### Structural mechanism for silencing of RXR by TR

**Figure 3:**
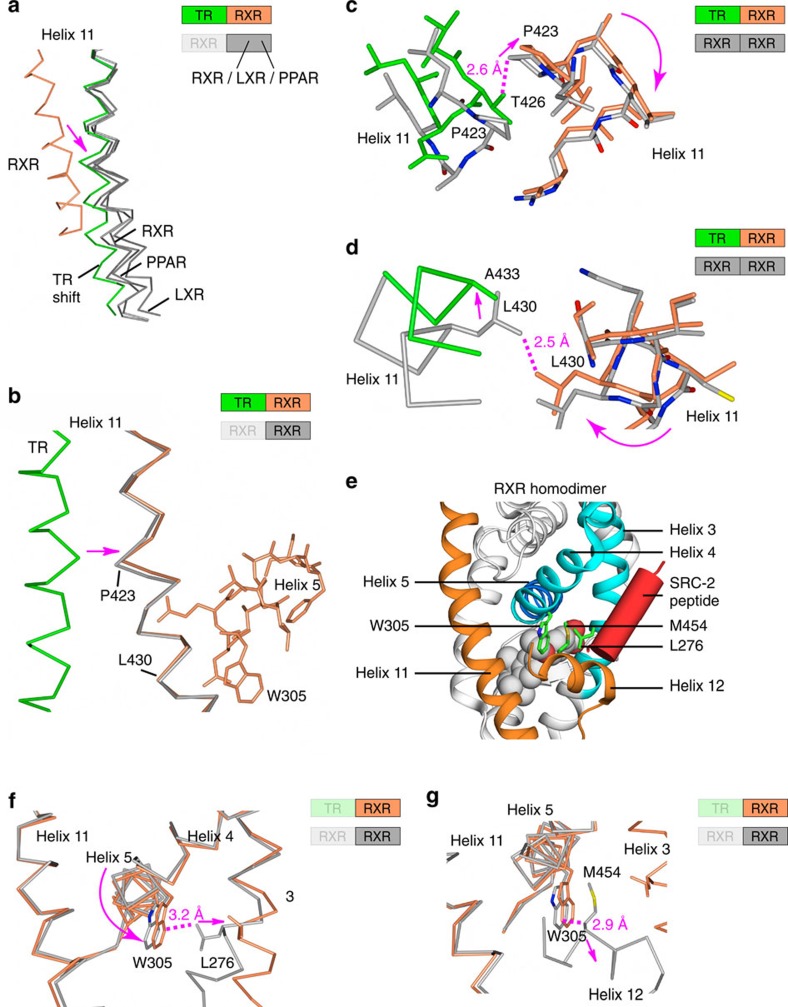
TR alters apo-RXR conformation via the helix 11 portion of the dimer interface. (**a**) Helix 11 of the dimer interface shown as Cα traces for TRβ·T3·SRC-2/apo-RXRα, coloured green and coral, respectively. The RXR homodimer (PDB 1MVC), RXR/PPARγ (PDB 1FM6) and RXR/LXR (PDB 1UHL) heterodimers were superimposed on the TRβ·T3·SRC-2/apo-RXRα structure using the RXR promoter molecule and are coloured grey. (**b**–**d**) The active conformation RXR homodimer (PDB 1MVC) superimposed on the TRβ·T3·SRC-2/apo-RXRα structure via RXR and coloured as in **a**, with the RXR homodimer coloured grey. (**b**) TR helix 11 (green) induces a shift in the RXR helix 11 (coral) relative to the RXR homodimer (grey). This shift is adjacent to RXR helix 5. (**c**) The unique position of TR T426 in helix 11 induces a shift in RXR P423 in helix 11 and a rotation of the RXR helix 11 backbone. (**d**) The location of TR A433 in helix 11 away from the dimer interface compared with the equivalent residue in RXR, L430, allows RXR L430 and the RXR helical backbone to rotate in context of the TRβ·T3·SRC-2/apo-RXRα heterodimer. (**e**) RXR in the active conformation (PDB 1MVC) with RXR ligand (MBS649) shown as space filled and SRC-2 peptide bound to the AF-2 co-activator-binding surface coloured red. RXR W305 in helix 5 mediates contacts with the ligand, M454 in helix 12 and the co-activator-binding site via L276 in helix 3. Colour is used to help differentiate secondary structural elements and provide depth for overlapping elements; helix 3 and 4 in cyan, helix 5 in blue, and helix 10/11 and helix 12 in orange. (**f**) TRβ·T3·SRC-2/apo-RXRα was superimposed with the active conformation RXR homodimer (PDB 1MVC) and shown as Cα trace. The rotation of helix 5 in TRβ·T3·SRC-2/apo-RXRα repositions W305 such that it clashes with the active conformation of RXR L276. (**g**) Same as **f**, but showing the active conformation of helix 12, and the clash with the rotated position of W305 in the TRβ·T3·SRC-2/apo-RXRα heterodimer.

The TRβ·T3/apo-RXRα crystal structure revealed that the structural basis of RXR silencing is via an allosteric signal emanating from the middle of the dimer interface. Compared with other RXR dimers fully occupied by ligands—including the RXRα homodimer^46^, and permissive RXR heterodimer complexes with LXR^47^ and PPARγ^48^—our structure of the TRβ·T3/apo-RXRα heterodimer shows a marked shift in TRβ helix 11 (Fig. 3a). This shift induces a rotation of RXRα helix 11, which is visualized by comparing our TRβ·T3/apo-RXRα heterodimer with the RXRα homodimer (Fig. 3b–d) or with the apo-RXRα tetramer (Supplementary Fig. 3). In the N terminus of helix 11, TRβ T426 is shifted towards RXRα P423 (Fig. 3b), which shows a rotation away from TRβ in the heterodimer structure (Fig. 3c, Supplementary Fig. 3a). Towards the C terminus of helix 11, TRβ is shifted away from RXRα, leading to a further rotation of RXRα helix 11 to maintain van der Waals contacts between RXRα L430 and TRβ helix 11 (Fig. 3d, Supplementary Fig. 3b).

Notably, the TRβ-directed rotation of RXRα helix 11 in the TRβ·T3/apo-RXRα structure induces a corresponding rotation of the adjacent RXR helix 5, which in turn disrupts the active conformation of RXRα. In the active conformation of the RXR homodimer, W305 in helix 5 mediates contacts with the bound ligand, M454 in helix 12 and L276 in helix 3, which is part of the AF-2 co-activator-binding surface (Fig. 3e). In contrast, in the TRβ·T3/apo-RXRα structure TRβ-induced rotation of RXRα helix 5 in the heterodimer provokes a clash with the active conformation of helix 12 that pushes both L276 in helix 3 and M454 in helix 12 away from W305 in helix 5 (Fig. 3f,g). Importantly, rotation of helix 5 is not observed in the apo-RXRα homotetramer (Supplementary Fig. 3c), and is thus not a consequence of the substantial shift in helix 3 that is observed in both the TRβ·T3/apo-RXRα and apo-RXRα tetramers, which is rather determined by the tetramer packing. The electron density map allows the clear visualization of the main chain rotation required for interpretation of this data (Supplementary Fig. 3d,e), and the rotation of helix 5 is significant (Supplementary Fig. 4). Thus in the TRβ·T3/apo-RXRα structure the TRβ-induced rotation of RXRα helix 11 and helix 5 disables the active conformation of RXRα.

### Structural role of SCA co-evolved amino acids

Work from the Mangelsdorf and Ranganathan labs^10^ identified a network of co-evolved amino acids that are energetically coupled and mediate allosteric signalling in RXR heterodimers. A SCA was used to identify a network of 27 amino acids that comprise an allosteric signalling network for communication between RXR and its heterodimer partner. Importantly, an extensive mutagenesis screen showed that mutation of residues in one molecule allosterically impacted ligand response (that is, permissivity) from the partner^10^, although the structural mechanism that drives this effect at the atomic level remained unknown.

**Figure 4:**
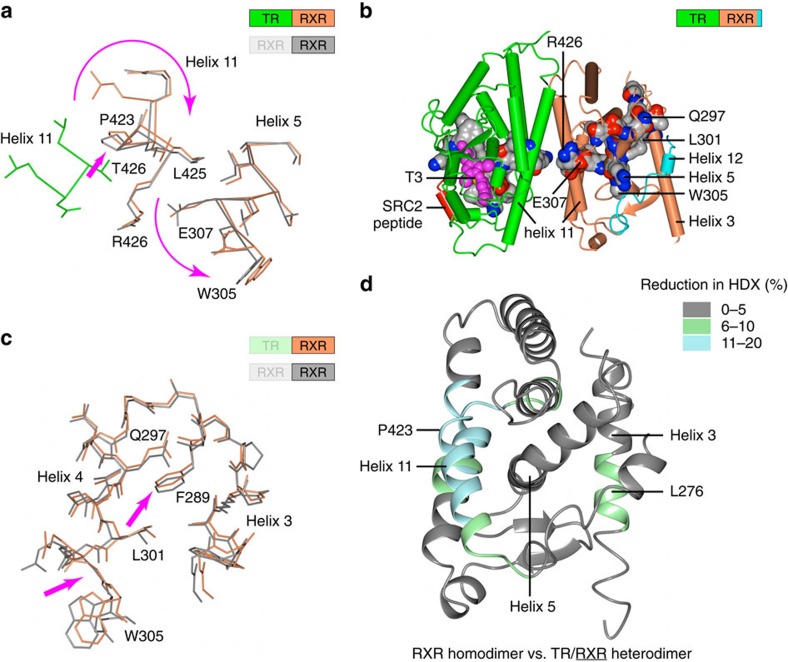
Co-evolved amino acid network mediates structural allostery between RXR and TR. (**a**) The TRβ·T3·SRC-2/apo-RXRα structure (green and coral) superimposed with the active conformation RXR homodimer (PDB 1MVC) via RXR and shown in grey. RXR residues in helix 11 (L425, R426) and helix 5 (W305, E307) are part of a network of co-evolved amino acids identified using a statistical coupling analysis (SCA). (**b**) The SCA network amino acids shown as space filled on the TRβ·T3·SRC-2/apo-RXRα heterodimer link the dimer interface, ligand-binding pocket and AF-2 co-activator-binding site. (**c**) Active conformation RXR homodimer (PDB 1MVC) superimposed with RXR from the TRβ·T3·SRC-2/apo-RXRα heterodimer. Shown are helix 3 and helix 4 of the AF-2 surface, and W305 in helix 5. The rotation of helix 5 induces an altered conformation of the AF-2 surface via the SCA network amino acids. (**d**) Regions in RXR that are protected from HDX on heterodimerizaton with TR.

Helix 5 lies at the core of the SCA network, and connects the dimer interface, the ligand-binding pocket and the co-activator-binding site (Fig. 4a,b). This network includes residues in the core of RXRα that promote rotation of helix 5 and subsequent silencing of RXRα, including residues in helix 11 (for example, L425 and R426), helix 5 (for example, E307 and W305) and helix 3 (for example, L276). RXR R426A and W305A mutants, and the analogous mutants in a permissive RXR heterodimer partner afford a dramatic loss-of-function equivalent to helix 12 deletion^10^. However, while RXR E307A (helix 5) has a modest effect on function, its analogous mutation in a permissive RXR heterodimer partner blunts the permissive response with RXR ligand. The rotation of RXRα W305 observed in the TRβ·T3/apo-RXRα structure would directly impact ligand binding, thereby accounting for the lower affinity of the TR/RXR heterodimer for RXR ligands^34^. The importance of these residues is underscored by the transmission of helix 5 rotation to the co-evolved amino acid residues in helix 3 and helix 4 at the core of the co-activator-binding site (Fig. 4c). Our structural data suggests a model where this co-evolved network controls the rotation of helix 5, thus impacting the dimer interface, and the ligand- and co-activator-binding sites.

In our TRβ·T3/apo-RXRα heterodimer structure, RXRα ligand binding would require RXRα helix 3 to move back into the agonist conformation, driving RXRα L276 on helix 3 towards RXRα W305 in helix 5, leading to a reversal of the TRβ-induced rotation of RXRα helix 5. To determine if the conformation of these RXRα regions are affected by TRβ heterodimerzation, we performed differential HDX mass spectrometry comparing RXRα in its homodimeric state versus heterodimerized to TRβ. In the presence of T3, 9cRA and co-activator peptide, the secondary structural elements of TRβ-bound RXRα that were protected from amide exchange centred around P423 in helix 11 and extended to L276 in helix 3, relative to RXRα in the homodimer (Fig. 4d and Supplementary Table 1). The changes in HDX support our model where these regions direct allosteric signalling within the heterodimer resulting in the silencing of RXR by TR.

**Figure 5:**
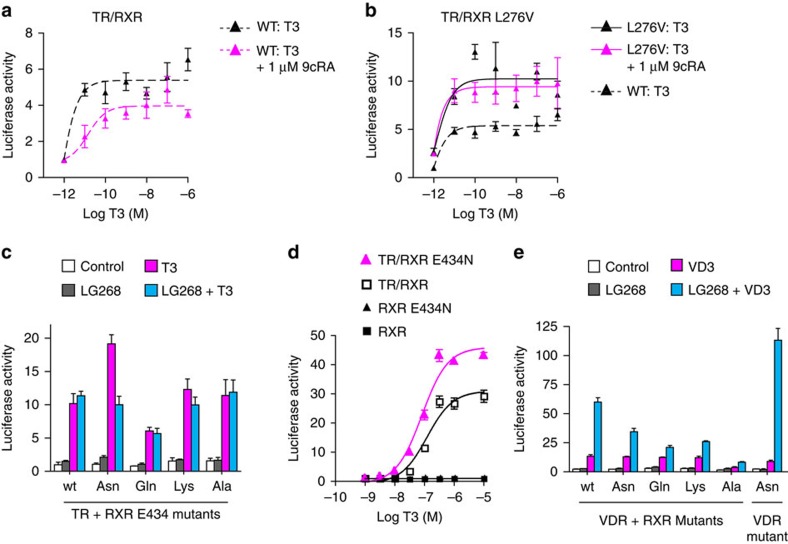
Mutagenesis confirms a structure-function role for co-evolved amino acids. (**a**,**b**) CV-1 cells co-transfected with TRβ expression plasmid, (**a**) RXRα or (**b**) RXRα L276V mutant expression plasmid, and a TR-responsive luciferase reporter. Cells were treated with vehicle, or the indicated dose of T3±1 μM 9cRA for 24 h. (**c**) Drosophila SL2 cells co-transfected with TRβ expression plasmid, RXRα or RXRα E434-mutant expression plasmid, a TR-responsive luciferase reporter. Cells were treated with vehicle, the RXR agonist LG100268 (LG268; 100 nM) and/or 1 μM TR agonist (T3) for 24 h. (**d**) CV-1 cells co-transfected with TRβ expression plasmid, RXRα or RXRα E434N mutant expression plasmid, and a TR-responsive luciferase reporter. Cells were treated with vehicle or the indicated dose of T3 overnight. (**e**) Drosophila SL2 cells co-transfected with VDR expression plasmid, RXRα or RXRα E434-mutant expression plasmid, and a VDR-responsive luciferase reporter. Cells were treated with vehicle, the RXR agonist LG100268 (LG268; 100 nM) and/or 1 μM VDR agonist (vitamin D3) for 24 h. Luciferase activity is shown normalized to vehicle-treated cells and was performed in quadruplicate; plotted with the average±s.e.m and representative of at least three experiments.

To further test the role of helix 5 rotation in connecting the dimer interface with helix 3, we introduced the RXRα helix 3 mutation L276V, as we hypothesized that the smaller valine residue at this site (Fig. 3f) would facilitate packing of helix 3 against the rotated helix 5, even in the presence of RXR ligand. With the wild-type TRβ/RXRα heterodimer, 9cRA impaired T3-mediated induction of a TR-responsive luciferase reporter in CV-1 mammalian cells transfected with TRβ/RXRα, shown by a highly significant effect of 9cRA in a two-way analysis of variance (ANOVA, F(1,42)=27; *P*<0.001). There was also a trend towards an interaction between the T3 dose and 9cRA terms, suggesting a potential effect of 9cRA on making T3 less potent (ANOVA, F(6,42)=2; *P*=0.071). (Fig. 5a). In contrast, the mutated TRβ/RXRα L276V heterodimer showed a gain-of-function in response to T3 (10- versus 5-fold activation), (ANOVA, WT+T3 versus L276V+T3: L276V effect F(1,42)=67; *P*<0.0001) and this mutation abolished the inhibitory effect of 9cRA on TR activation (Fig. 5b). These gain-of-function results are consistent with our model, where the interaction between RXRα W305 in helix 5 and L276 in helix 3 contributes towards the allosteric silencing of RXR by TR.

### Role of helix 11 C terminus in cross-dimer signalling

We also tested the role of helix 11 in modulating the allosteric signal. While our studies here point to roles for the N-terminal region of helix 11 in TRβ and RXRα in rotating helix 5, we previously noted that the C terminus of helix 11 could be differentially positioned by distinct ligands, thereby controlling the packing of helix 12 in the agonist conformer. This modulation of helix 11 provides the structural basis for partial agonist activity on NRs, by titrating the dynamics or stability of helix 12 as it forms the active conformer^22^,^23^. Above we also noted that RXRα E434 can form hydrogen bonds across the dimer interface with some heterodimer partners^13^, right in the plane of the ligands, suggesting a conduit for structural information from the ligand to the partner receptor. In total, this suggests that the position of RXRα helix 11 can be affected by the dimer partner and modulated by either the RXR ligand or partner ligand. Although the RXR E434 side chain is not visible in our structure, it would be positioned to potentially interact with TRβ S437 in helix 11. We previously demonstrated a role for this hydrogen bond in signal integration between RAR/RXR heterodimers^21^. Mutation of RXRα E434 to asparagine induced a gain-of-function in response to T3, both in drosophila SL2 cells that lack endogenous RXR and TR (Fig. 5c) and in CV-1 monkey cells (Fig. 5d). Thus, the C terminus of RXRα helix 11 plays a critical role in regulating the response of the TRβ/RXRα heterodimer to T3.

To extend these results, we performed similar experiments with VDR, which is another heterodimer partner that silences RXR. Our HDX analysis of the full-length VDR/RXRα heterodimer with various combinations of ligands, DNA and co-activators established that vitamin D3 ligand induces stabilization of the same RXRα regions found here to be affected by TRβ, including helix 3 and helix 11 (ref. 7). We therefore tested a series of RXRα E434 mutations in complex with VDR, and a mutation of the corresponding residue in VDR, K395 (Fig. 5e). Although RXR ligand has no activity on its own, within the context of the VDR/RXRα heterodimer it is conditionally permissive because it enhances vitamin D3-induced transactivation. The helix 11 mutations selectively modulate the conditional activation by the combination of vitamin D3 and RXR agonists, and lead to both gain- and loss-of-function. Taken together, these data suggest that similar helix 11-mediated mechanisms control allosteric signalling across the dimer interfaces of TR/RXR and VDR/RXR heterodimers, and that there are several mechanisms for heterodimer signal integration.

### Ligand signalling in PPARγ/RXRα employs SCA network

**Figure 6:**
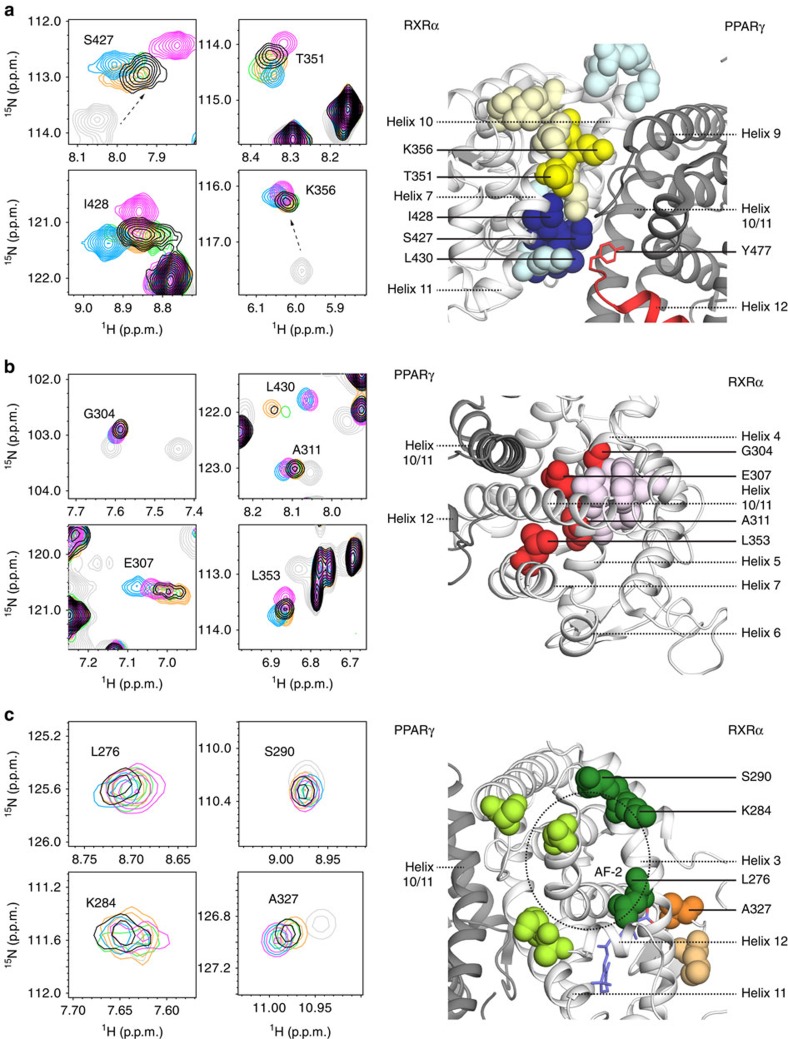
NMR reveals ligand binding to PPARγ affects the conformation of RXR. NMR data are coloured grey for 9cRA-bound RXRα; black for 9cRA-bound RXRα heterodimerized to apo-PPARγ or the same bound to the following PPARγ ligands: rosiglitazone (magenta), MRL20 (blue), MRL24 (orange) or SR1664 (green); plotted on PPARγ/RXRα (PDB 1FM9). (**a**) NMR data (left) focusing on residues in RXRα helix 7 and helix 10/11 dimer interface that are perturbed by ligand binding to PPARγ, which are plotted onto the PPARγ/RXRα crystal structure and coloured according to structural location (yellow for helix 7; blue for helix 10/11); coloured dark if shown in the NMR data to the left or light if not. (**b**) NMR data (left) focusing on residues in core of RXRα that are perturbed by ligand binding to PPARγ, which are plotted onto the PPARγ/RXRα crystal structure and coloured red; and coloured dark if shown in the NMR data to the left or light if not. (**c**) NMR data (left) focusing on residues in RXRα helix 12, the AF-2 surface and the ligand-binding pocket that are perturbed by ligand binding to PPARγ, which are plotted onto the PPARγ/RXRα crystal structure and coloured according to structural location (green for AF-2/helix 12; orange for the ligand-binding pocket); coloured dark if shown in the NMR data to the left or light if not.

To determine if ligand-selective signalling can occur between LBDs in RXR heterodimers, we took advantage of our extensive structural and chemical biology efforts with PPARγ to compare ligands that produce different signalling outcomes or graded receptor activation^4^,^25^,^26^,^27^,^49^. We performed NMR analysis using isotopically labelled RXRα and unlabelled PPARγ to observe conformational changes in RXRα resulting from ligand binding to PPARγ. We added several PPARγ ligands to the apo-PPARγ/9cRA·RXRα complex (Supplementary Fig. 5); including a full PPARγ agonist (rosiglitazone), a near full agonist (MRL20), a partial agonist (MRL24) and an antagonist/non-agonist (SR1664). Relative to the other liganded states, PPARγ full agonists rosiglitazone and MRL20 caused notable NMR chemical shift changes for RXRα residues at the core of the dimer interface (Fig. 6a,b and Supplementary Fig. 6), including residues in helix 11 (for example, S427, I428, G429 and L430) and helix 7 (for example, T351 and K356). Other more modest NMR resonance shifts are observed in the RXRα dimer interface, including residues at the N terminus of helix 10/11 (for example, Q411 and G413). Importantly, the PPARγ full agonist-induced NMR chemical shift changes for these residues at the core of the dimer interface were less prominent for the partial agonist and antagonist/non-agonist, suggesting these RXRα residues are structural sensors for PPARγ ligand activity. Thus, the RXRα dimer interface responds to PPARγ ligands in a manner that tracks with the ligand pharmacology, ranging from full agonist to non-agonist.

A possible mechanism for this allosteric communication through the RXRα dimer interface involves the C terminus of PPARγ helix 12. We previously demonstrated that PPARγ agonists, but not partial agonists or antagonists, stabilize helix 12 of PPARγ^4^,^25^,^26^,^27^. In the PPARγ/RXRα heterodimer crystal structure^50^, RXRα K431 in helix 11 forms a hydrogen bond with PPARγ Y477, the most C-terminal residue in PPARγ. RXRα residues affected differently by the graded PPARγ ligands are structurally close to this region. Thus, the effect of PPARγ full agonists on RXRα residues in the dimer interface are likely mediated through stabilization of PPARγ helix 12 and its interaction with RXRα.

However, structural perturbations also penetrate into other regions of RXRα, including the hydrophobic core, ligand-binding pocket and the AF-2 surface. This includes effects on RXRα core residues in helix 5 and helix 7 (for example, G304, E307, A311 and L353) (Fig. 6b and Supplementary Fig. 6). One of two tryptophan residues in the RXRα LBD core (W305 or W282) is also affected. Helix 5 sits between helix 11 and helix 3 at the nexus of the ligand-binding pocket and the AF-2 surface. The perturbed residues provide a direct connection to structural changes in helix 3, the AF-2 surface and the ligand-binding pocket (Fig. 6c and Supplementary Fig. 6). This includes small, but notable changes in RXRα helix 3 residue L276, as well as AF-2 surface residues K284 and S290, which are part of a region that forms electrostatic interactions with the bound co-activator peptide in the crystal structures. Thus, RXRα helix 5 is a key part of the LBD core that transmits PPARγ ligand-induced allosteric signals from the dimer interface to the RXRα ligand-binding pocket and the AF-2 surface.

An additional region of the RXRα LBD core, helix 9, also mediates PPARγ ligand-induced allosteric signalling across the dimer interface in a similar direction. Helix 9 forms part of the core that contacts the dimer interface, and stabilizes the AF-2 surface via interaction with helix 3 and helix 4 residues. PPARγ ligand-induced NMR chemical shift changes in RXRα helix 11 (for example, Q411 and G413) and helix 9 (for example, G368), which lie in this region, suggest that helix 9 may also transmit allosteric information from the dimer interface to the AF-2 surface. Additional NMR resonances showing specific changes in response to PPARγ ligands include A457 at the C terminus of RXRα helix 12 and A327 in the RXRα ligand-binding pocket.

All together, our NMR data reveal that binding of different ligands to its heterodimer partner, PPARγ, can cause subtle but significant changes in the conformation of RXRα. Using HDX mass spectrometry, we confirmed that ligand binding to PPARγ imparts structural changes in RXRα (Supplementary Fig. 7 and Supplementary Table 2). These effects are not only present at the dimer interface, but also extend through the core of the RXRα LBD, to the AF-2 surface, helix 12 and the ligand-binding pocket. As we discuss below, these structural regions involve a network of co-evolved amino acids in NRs, which are energetically coupled and mediate allosteric signalling in RXR heterodimers.

### Conservation of allosteric signalling through helix 5

**Figure 7:**
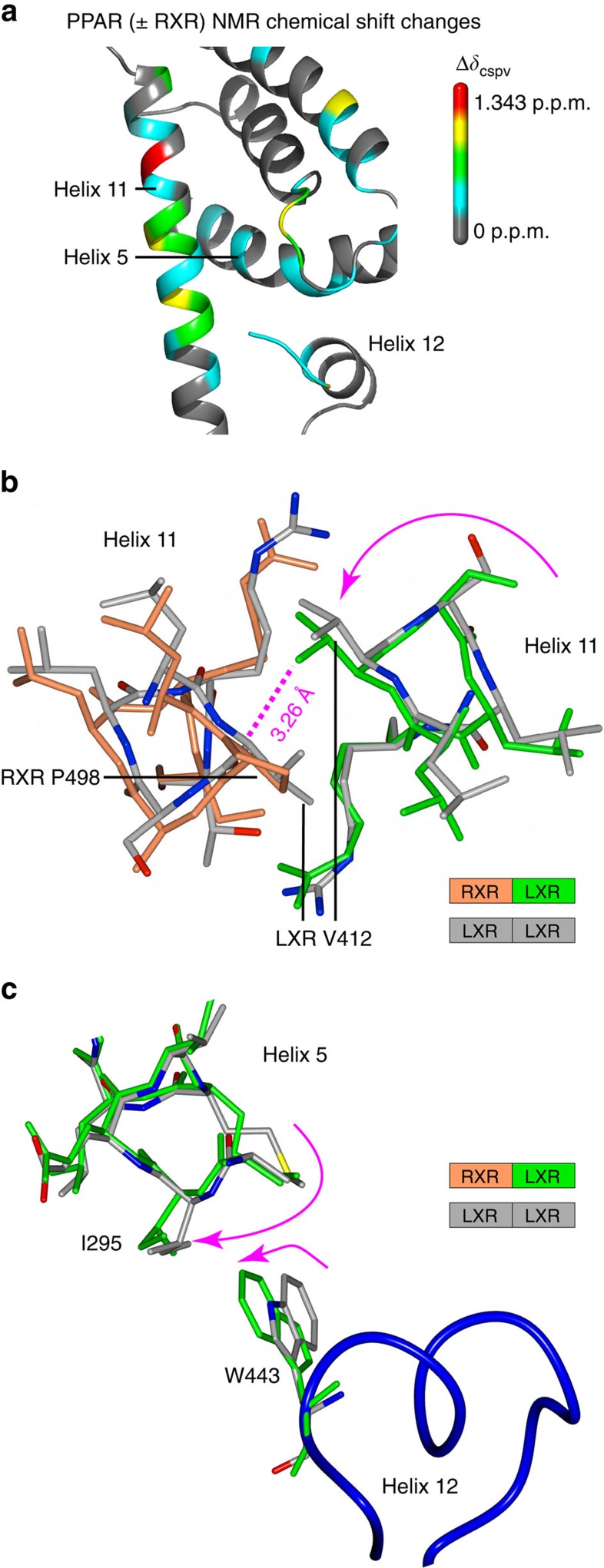
Co-evolved amino acid network with other receptors. (**a**) NMR chemical shift perturbations in PPARγ on heterodimerization with RXRα mapped onto the structure of the PPARγ LBD (PDB 2PRG). (**b**) LXR homodimer (PDB 3IPU) coloured grey and superimposed with the LXR promoter from the LXR/RXR heterodimer (PDB 1UHL) coloured green and coral, respectively, shows that RXR induces rotation of LXR helix 11. (**c**) The RXR-induced shift in LXR helix 11 (PDB 1UHL) induces a rotation of LXR helix 5 relative to the LXR homodimer (PDB 3IPU), allowing W443 in helix 12 to adopt an alternative conformation with greater van der Waals contacts and increased buried surface area.

Our data support a model where TR-induced rotation of RXR helix 5 drives TR silencing of RXR, and where rotation in the other direction drives the inhibitory effects of 9cRA on TR via the dimer interface. We propose that the co-evolved amino acid network lies at the core of this allosteric mechanism, which is consistent with our mutagenesis screen showing that these co-evolved amino acids impact allosteric signalling in RXR heterodimers^10^. Indeed, when we calculated NMR chemical shift differences between PPARγ as a monomer versus heterodimerized to RXRα^51^, structural perturbations were observed in the regions of the evolutionarily conserved residues in helix 11 and helix 5 induced by heterodimerization with RXRα (Fig. 7a).

If an evolutionarily conserved allosteric network directs helix 5 rotation, then it should manifest for other NRs. A comparison of the structures of the LXR homodimer with the permissive LXR/RXR heterodimer in the presence of co-activator peptide also shows that RXR induces a shift in LXR helix 11 that is transmitted through helix 5, again via the co-evolved amino acid network (Fig. 7b,c). This shift accommodates a flip of LXR W443 in helix 12 into a position against I295 in helix 5 (Fig. 7c) with additional van der Waals interactions and greater buried surface area, thereby stabilizing LXR helix 12 in the agonist conformation. Thus, RXR-induced rotation of LXR helix 5 is also the mechanism through which RXR drives co-activator binding to apo-LXR, a previously unexplained allosteric phenomena in heterodimer signalling^52^. This mechanism is also operational for heterodimers versus monomers of the RAR and the constitutive androstane receptor (Supplementary Fig. 8), supporting a general role for helix 5 rotation in allosteric control of RXR heterodimers. Thus, helix 5 rotation and the evolutionarily conserved SCA network of amino acid residues provide a structural conduit for signalling from the dimer partner, through helix 11, to the ligand-binding pocket and co-regulator-binding surface.

## Discussion

While originally conceived as an on/off switch in transcriptional regulation, it is now clear that NRs contain a number of allosteric fine-tuning mechanisms that allow a full range of graded signalling outcomes. NRs can be viewed more generally as dynamic scaffold proteins, where post-translational modifications and interaction with ligands, co-regulators and DNA modify the nature of the scaffold and the signalling outcomes^5^,^11^. A large body of work has described functional interactions between NR domains and these interacting molecules, which in sum define a NR signalling code^2^,^14^. Several studies have investigated various aspects of permissiveness in RXR heterodimers^33^,^53^,^54^,^55^. However, most of the structural features for allosteric signal integration have remained a mystery, limited in part by our insufficient structural understanding of signalling within the individual domains.

**Figure 8:**
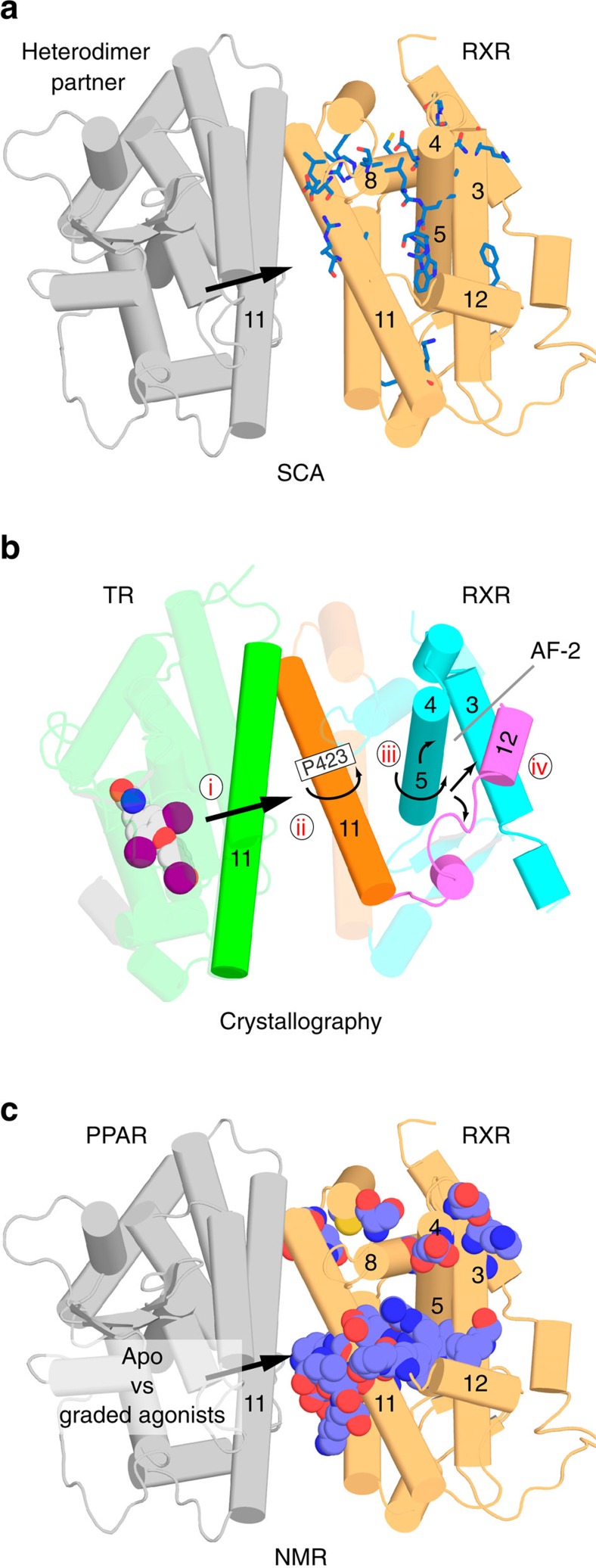
Summary of structural studies and allostery in RXR heterodimers. (**a**) The SCA network residues plotted on RXR using the PPARγ/RXRα heterodimer structure as a model (PDB 1FM6). (**b**) Schematic diagram summarizing our TRβ·T3·SRC-2/apo-RXRα crystal structure showing how TR structurally silences RXR. The signal that emanates from TR (i) induces a shift in RXR helix 11 (ii), leading to a rotation of helix 5 (iii) resulting in structural arrangements that cause RXR helix 12 to adopt an inactive conformation (iv). (**c**) Summary of residues affected in the NMR analysis of ligand-selective signalling in PPARγ/RXRα, plotted on PDB 1FM6. Helix numbers are indicated for elements of interest. Arrows indicate the flow of the allosteric signal.

Here we used a variety of structural and functional approaches to show how the dimer partner controls the permissivity, or activity, of RXRα in the integration of two distinct ligand-regulated receptors into a single transcriptional response using residues comprising the SCA network (Fig. 8a). Using NMR and crystallography, we show that structural differences in RXRα affected by the different dimer partners, TRβ and PPARγ, initiate distinct allosteric signals that suppress or permit modulation of heterodimer activity through RXRα. These signals are transmitted through amino acid residues including the co-evolved network previously identified by SCA^10^. Our NMR data reveal that in the presence of RXR agonist, dimerization with apo-TRβ triggers a considerable change that reinstates an RXRα conformation that exchanges between two or more conformations on the μs–ms timescale. When compared with activated RXR homodimer^46^ and permissive RXR heterodimer structures, LXR/RXR^47^ and PPAR/RXR^48^, our TRβ·T3/apo-RXRα crystal structure suggests that dimerization with the non-permissive partner TRβ rotates RXRα helix 11, twists helix 5, drives open the ligand-binding pocket and induces the apo conformer of RXRα (Fig. 8b). This may account for the observation that TR lowers the affinity of RXRα for its ligand^34^. However, this conformational relay mechanism also operates in reverse, mediating the intrinsic activity of the apo-LXR/RXR heterodimer^52^. Here binding of RXR to LXR leads to a compensatory rotation of LXR helix 5, which directly stabilizes LXR helix 12 in the active conformation. Our studies reveal helix 5 as a central locus for allosteric control between the dimer interface, helix 12, ligand-binding pocket and AF-2 surface. The helices that comprise the SCA network act like a set of interlocking gears to integrate information from the functionally important sites in the NR LBD.

We identified a number of distinct routes for signal transduction through the dimer interface. For example, in our NMR studies of ligand-selective signalling in PPARγ/RXRα, only full agonists of PPARγ, which stabilize helix 12 in the agonist conformation, induce significant alterations in the RXRα dimer interface adjacent to the C terminus of PPARγ helix 12. In this way, RXRα is able to ‘feel’ the position of the partner helix 12 and the degree of partner agonist activity. These types of cross-dimer interactions may also help stabilize helix 12 of the heterodimer partner in the active conformation, as previously suggested^48^. A second set of RXRα regions affected by all of the various PPARγ liganded states include the hydrophobic core, helices 8–10 and helices 3–4 of the AF-2 surface (Fig. 8c). These regions in general employ the network of co-evolved residues predicted by SCA. Of these, PPARγ full agonists appear to cause a more prominent effect, but the specific role of this structural conduit is not clear. It could mean that PPARγ full agonists may provide additional stabilization to the RXRα AF-2 surface, or alter the shape of the AF-2 to give preferences for certain co-activators. Our NMR data further suggests that this interlocking relay system is also modulated by the ligand, as one of the two tryptophan residues in the RXR LBD, W305 in helix 5 or W282 in helix 3, was differentially sensitive to PPAR ligands. We thus envision that structural elements in helix 5 of RXR and the dimer partner can move in a coordinated way with the C-terminal region of the helix 11 dimer interface to coordinate both receptor- and ligand-specific signals into an integrated transcriptional response with the co-evolved amino acids playing a primary role.

We identified the C terminus of helix 11, adjacent to the bound ligands, as also contributing to heterodimer signalling. Within each monomer, there are two known mechanisms through which different ligands can produce a range of signalling outcomes from full agonist to antagonist. One is by direct modulation of helix 12, where the physical contact between ligand and helix 12 determines the percentage of time the active conformation of helix 12 is stabilized, docked across helix 3 and helix 11 to form the AF-2 surface. A second mechanism—indirect modulation—occurs when the ligand can position helix 11 so as to provide suboptimal van der Waals packing with helix 12, and thus indirectly control its stability in the agonist conformation^21^,^22^,^23^,^24^. Our data suggest an extension of this model where the position of helix 11 is also controlled by the heterodimer partner helix 11. Our mutagenesis data further suggests that the C terminus of helix 11 is also positioned by the type of dimer partner, in addition to the specific ligand, contributing to permissive versus non-permissive heterodimer signal integration.

## Methods

### Protein expression, purification and ligands

Human RXRα LBD (amino acids 223–462), human TRβ LBD (residues 202–461) and human PPARγ LBD (residues 203–477; isoform 1 numbering) were cloned into a pET vector with a ligation-independent cloning site as TEV-cleavable hexahistidine-tagged (His-tag) fusion proteins. RXRα LBD was induced in BL21(DE3) cells, and purified with immobilized nickel affinity chromatography. The eluted protein was mixed with a 1:30 ratio (by mg weight) of His-tag TEV protease and dialysed overnight in 20 mM Tris pH 8, 50 mM NaCl, 50 mM β-mercaptoethanol and 10% glycerol. The protein solution was again purified using immobilized nickel affinity chromatography to remove uncut protein, the cut His-tag and the TEV protease. The flow through was diluted 2 × in H_2_O and subjected to gel filtration in buffer consisting of 20 mM Tris 8.0, 50 mM NaCl, 10% glycerol and 5 mM β-mercaptoethanol. TRβ LBD was induced in BL21(DE3)Rosetta cells, and purified with immobilized nickel affinity chromatography (Qiagen) in a manner identical to that of RXRα. For crystallography, purified TRβ LBD that had not been subjected to TEV proteolysis was incubated with purified RXR lacking a His-tag (at a ratio of 2:1 RXR to TR). The complex was purified with immobilized nickel affinity chromatography using buffers and gradients as described above and then the TR LBD His-tag was removed by proteolysis with TEV protease overnight while the complex was being dialysed to 20 mM Tris 8.0, 150 mM NaCl, 5 mM BME and 10% glycerol. PPARγ LBD was expressed and purified using similar methods, and final NMR sample conditions contained 20 mM KPO4 (pH 7.4) and 50 mM KCl^56^. Ligands were purchased from commercial sources, or in the case of MRL20, MRL24 and SR1664 were synthesized^27^,^57^.

### NMR spectroscopy and analysis

NMR data were collected at 298 K on a 700 MHz Bruker NMR instrument equipped with a conventional TXI triple resonance probe and on a 800-MHz Varian NMR instrument equipped with a cryogenically cooled triple resonance probe. Ligands that were added to proteins were dissolved in DMSO-d_6_. NMR experiments were performed using pulse sequences and standard experimental parameters provided with Bruker Topspin 3.0. RXRα LBD chemical shift assignments^37^ were validated and/or transferred to various complexed states using standard 2D and 3D NMR TROSY-based methods, including HSQC, HNCO, HNCA, HN(CO)CA and HN(CA)CB and ^15^N-NOESY-HSQC experiments. Data were processed using Bruker Topspin 3.0 or NMRPipe^58^ and analysed with NMRViewJ^59^. NMR chemical shift perturbations (Δ*δ*CSP) for PPARγ LBD in the monomer form and heterodimerized to RXRα LBD were calculated from published values^51^ as follows: Δ*δ*CSP=|Δ*δ*HN|+(0.154 × |Δ*δ*N|)+(0.341 × |Δ*δ*C′|); with Δ*δ*HN, Δ*δ*N and Δ*δ*C′ as the backbone ^1^H_N_, ^15^N and ^13^C′ (carbonyl) NMR chemical shift differences between monomer and heterodimer, respectively, and mapped onto the PPARγ LBD crystal structure (PDB 2PRG).

### Crystallization, structure determination and refinement

The TRβ·T3·SRC-2/apo-RXRα complex was formed by mixing a threefold molar excess of T3 (Sigma) and SRC-2 peptide (HKILHRLL). Crystal trials were initially conducted using commercially available sparse matrix screens, from which microcrystals were identified and subsequently streak seeded to produce a high resolution diffracting crystal. The well solution consisted of 20% PEG 3350, 100 mM Tris pH 8.0, 0.1 M ammonium acetate and 1 mM 11-Methoxy-3,7,11-trimethyl-2E,4E-dodecadienoic acid. The crystal was flash cooled with liquid nitrogen after briefly immersing in paratone-n as a cryo-protectant. Data was collected at SSRL beamline 11-1 at 100 K. The structure was solved with molecular replacement using Phaser^60^ using search models for TR (PDB 3GWS) and RXR (PDB 1G5Y). Initially scaled at 3.5 Å, the model was refined using PHENIX^61^ to an *R*_free_ of 33%. The data set was anisotropic and was therefore rescaled to 3.2 Å and truncated to 3.2 × 3.8 Å based on a 1.5 signal-to-noise (*σ*) cut-off using the UCLA Diffraction Anisotropy Server^62^. This allowed the refinement to lower the *R*_work_/*R*_free_ to 23.6/28.1%. The reported Rmerge for our structure is from the pre-truncated data set and was not used to determine the resolution cut-off. Instead, an anisotropic cut-off of 1.5*σ* was used based on recommendations of Brunger *et al.*^63^,^64^ to avoid discarding reflections when working with low resolution structures and with modern refinement practices that can accommodate lower signal reflections. Thus, the very good *R*_free_ and geometry statistics for this resolution likely reflect the use of higher resolution structures for molecular replacement. The TLSMD server was used to identify optimal TLS groups^65^. The model was rebuilt using COOT^66^. Structural figures were generated with CCP4MG^67^.

### HDX mass spectrometry

HDX was performed using a fully automated in-house system^49^,^68^ with some modifications. Briefly, protein samples are incubated with D_2_O-containing buffer at 4 °C for 10, 30, 60, 900 and 3,600 s. Following on-exchange, forward or back exchange was minimized and the protein was denatured by dilution with 25 μl of quench solution (0.1% v/v trifluoroacetic acid/TFA in 3 M urea). Samples were then passed through an immobilized pepsin column (prepared in-house) at 50 μl min^−1^ (0.1% v/v TFA, 15 °C) and the resulting peptides were trapped on a C_8_ trap column (Hypersil Gold, Thermo Fisher). The bound peptides were then gradient eluted (5–50% CH_3_CN w/v and 0.3% w/v formic acid) across a 2 × 50-mm C_18_ HPLC column (Hypersil Gold) for 5 min at 4 °C. The eluted peptides were then subjected to electrospray ionization directly coupled to a high resolution Orbitrap mass spectrometer; Exactive for TR/RXR or QExactive for PPARγ/RXR (Thermo Fisher Scientific). For TR/RXR measurements, 4 μl of 10 μM protein was diluted to 20 μl of D_2_O buffer. Following the prescribed on-exchange interval, the reaction was quenched with a cold 3-M urea solution containing 1% TFA and 50 mM TCEP. For PPARγ/RXR measurements, 10 μM of HIS-PPARγ LBD protein (20 mM KPO4 pH 7.4, 50 mM KCl) in complex with 10 μM FLAG-RXR LBD (20 mM KPO4 pH 7.4, 50 mM KCl) was preincubated with 1:2 molar excess of compound. About 5 μl of protein solution was mixed with 20 μl of D_2_O-containing buffer (20 mM KPO4 pH 7.4, 50 mM KCl) to initiate on-exchange. Following on-exchange, forward or back exchange was minimized and the protein was denatured by dilution with 25 μl of quench solution (0.1% v/v TFA in 3 M urea). HDX values are the average of three individual on-exchange experiments acquired in a random order. HDX data analysis was performed with ‘HDX Desktop’ for TR/RXR samples and ‘HDX Workbench’ for PPARγ/RXR^69^,^70^. Each HDX experiment was carried out in triplicate and the intensity-weighted average *m/z* value (centroid) of each peptide isotopic envelope was calculated. Data-dependent tandem mass spectroscopy was performed in the absence of exposure to deuterium for peptide identification in a separate experiment using a 60-min gradient. Peptides with a Mascot score of ≥20 were included in the peptide sets used for HDX.

### Cell culture and luciferase co-transfection assays

CV-1 cells (ATCC) were maintained in DMEM (Invitrogen) with 10% FBS charcoal/dextran –treated (Hyclone). Cells were transfected using Fugene HD (Roche) with a DR-4 luciferase reporter with expression plasmids for RXRα and TRβ. After 6 h, cells were passaged and transferred into 384-well plates. Ligands were added the next day and allowed to incubate overnight before processing for luciferase activity. An equal volume of Britelite (PerkinElmer) was dispensed and the luminescence was measured on an Analyst GT plate reader (PerkinElmer). Drosophila SL2 cells (ATCC) were maintained in Schneider Drosophila Medium (Gibco) containing 5% dextran–charcoal-stripped FBS and transfected at a density of 6,500 cells per well in 96-well plates by calcium phosphate co-precipitation. Co-transfection experiments included 50 ng of reporter plasmid, 20 ng of β-galactosidase expression plasmid, 15 ng of each receptor expression plasmid and PGEM carrier DNA to give a total of 150 ng of DNA per well of a 96-well plate. Cells were transfected for 8 h and were treated for 18 h before harvesting and determination of luciferase and β-galactosidase activity. Luciferase data were normalized to the internal β-galactosidase control and represent the mean of triplicate assays plus s.e. Fold induction values were calculated as ligand-induced relative luciferase units/control relative luciferase units and propagated errors were calculated. Experiments used human full-length RXRα, TRβ, PPARγ and VDR expression plasmids with the appropriate luciferase reporter plasmid^10^, including TREx2-luc (TR-luc), PPREx3-luc (PPAR-luc) or ADH-mSppx3-luc (VDR-luc). RXR mutant expression plasmids were generated using the Stratagene QuikChange Site-Directed Mutagenesis kit and verified by DNA sequencing. Statistical analyses were performed with Graphpad Prism.

## Additional information

**Accession codes:** Atomic coordinates and structure factors have been deposited in the Protein Data Bank with accession code 4ZO1.

**How to cite this article:** Kojetin, D. J. *et al.* Structural mechanism for signal transduction in RXR nuclear receptor heterodimers. *Nat. Commun.* 6:8013 doi: 10.1038/ncomms9013 (2015).

## Supplementary Material

Supplementary Information Supplementary Figures 1-8 and Supplementary Tables 1-2

## Figures and Tables

**Table 1:** Data collection and refinement statistics.

	**T3-bound TRβ/SRC-2/apo-RXRα**
*Data collection*	
Space group	P 31 2 1
Cell dimensions	
*a*, *b*, *c* (Å)	63.25, 63.25 225.81
*α, β, γ* (°)	90.00, 90.00, 120.00
Resolution (Å)	3.2 (3.31–3.22)*
*R*_merge_	0.09 (0.7)
*I*/*σI*	19.9 (1.46)
Completeness (%)	96.5 (86.2)
Redundancy	11.1 (5.0)
	
*Refinement*	
Resolution (Å)	3.2
No. of reflections	8,950
*R*_work_/*R*_free_	0.2365/0.2814
*No.* of *atoms*	
Protein	3,221
Ligand/ion	23
Water	0
*B*-factors	
Protein	132.9
Ligand	96.1
R.m.s. deviations	
Bond lengths (Å)	0.004
Bond angles (°)	0.65

R.m.s., root mean squared; RXR, retinoid X receptor.

^*^Data in parenthesis correspond to the highest resolution bin.
