# Supplementary material for: Structural mechanism for signal transduction in RXR nuclear receptor heterodimers
Source: Nat Commun. 2015 Aug 20;6:8013. doi: 10.1038/ncomms9013 (PMC4547401; doi:10.1038/ncomms9013)
Supplement: Supplementary Information — Supplementary Figures 1-8 and Supplementary Tables 1-2 [file ncomms9013-s1.pdf]

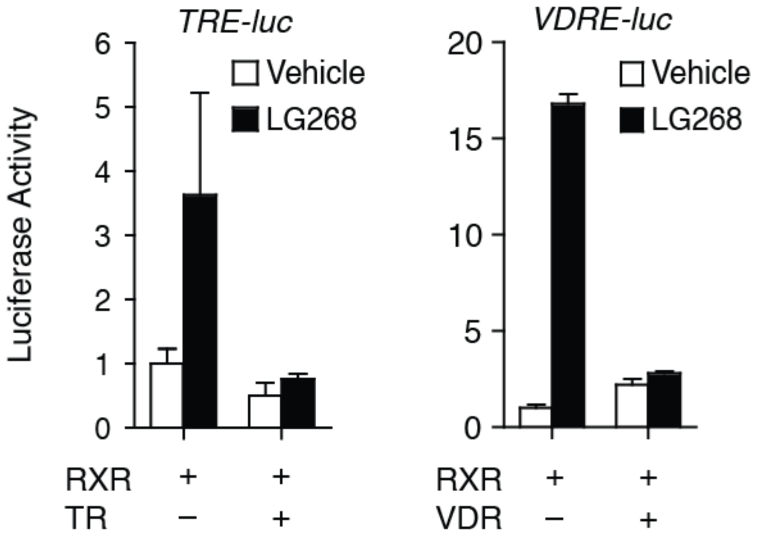


**Supplementary Figure 1. Permissive RXR heterodimer partners silence RXR ligand-induced transactivation.**

Drosophila S2 cells, which lack native RXR, TR or VDR, were transfected with the indicated expression plasmids and reporters, with RXR ligand (LG268) or the indicated TR or VDR ligand for 24 hours, then harvested for luciferase activity. Luciferase activity is shown normalized to vehicle treated cells and was performed in quadruplicate, plotted with the average (± s.e.m), and representative of at least 3 experiments.

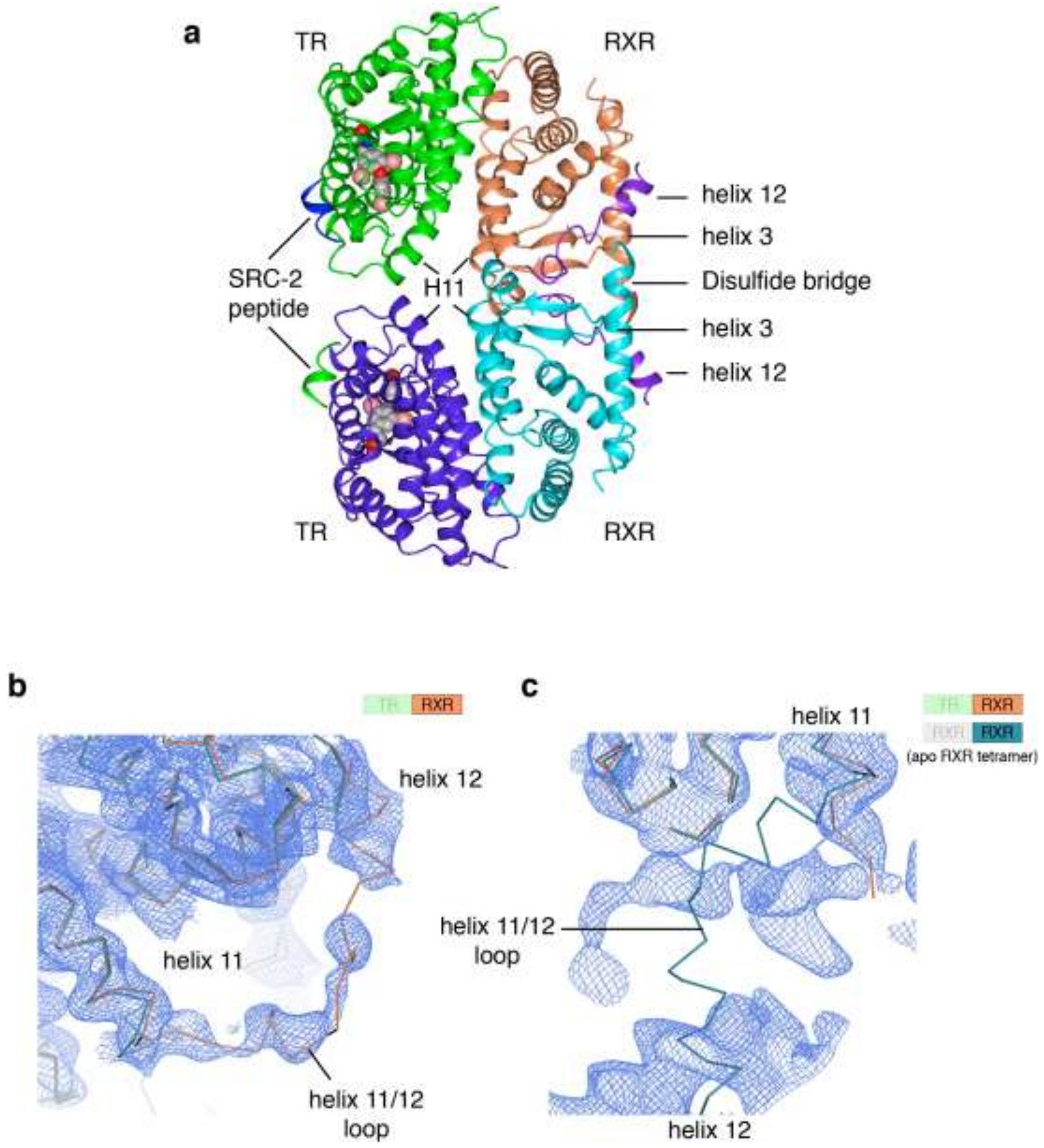


**Supplementary Figure 2. Crystal packing induced TR/RXR heterotetramer**

**(a)** Ribbon diagram of the TRβ•T3•SRC-2/apo-RXRα dimer, and the crystallographically related heterotetramer architecture.

**(b)** Positioning of the loop connecting helix 11 and 12 indicates a *cis*-binding helix 12 orientation in the new TR/RXR structure. The 2$F_o$-$F_c$ electron density map was contoured at $\sigma = 1.0$.

**(c)** The RXR tetramer structure (PDB 1G5Y) was superposed on the new TR/RXR structure (PDB 4ZO1) to illustrate the trans-binding helix 12 orientation. The 2$F_o$-$F_c$ electron density map was contoured at $\sigma = 1.0$.

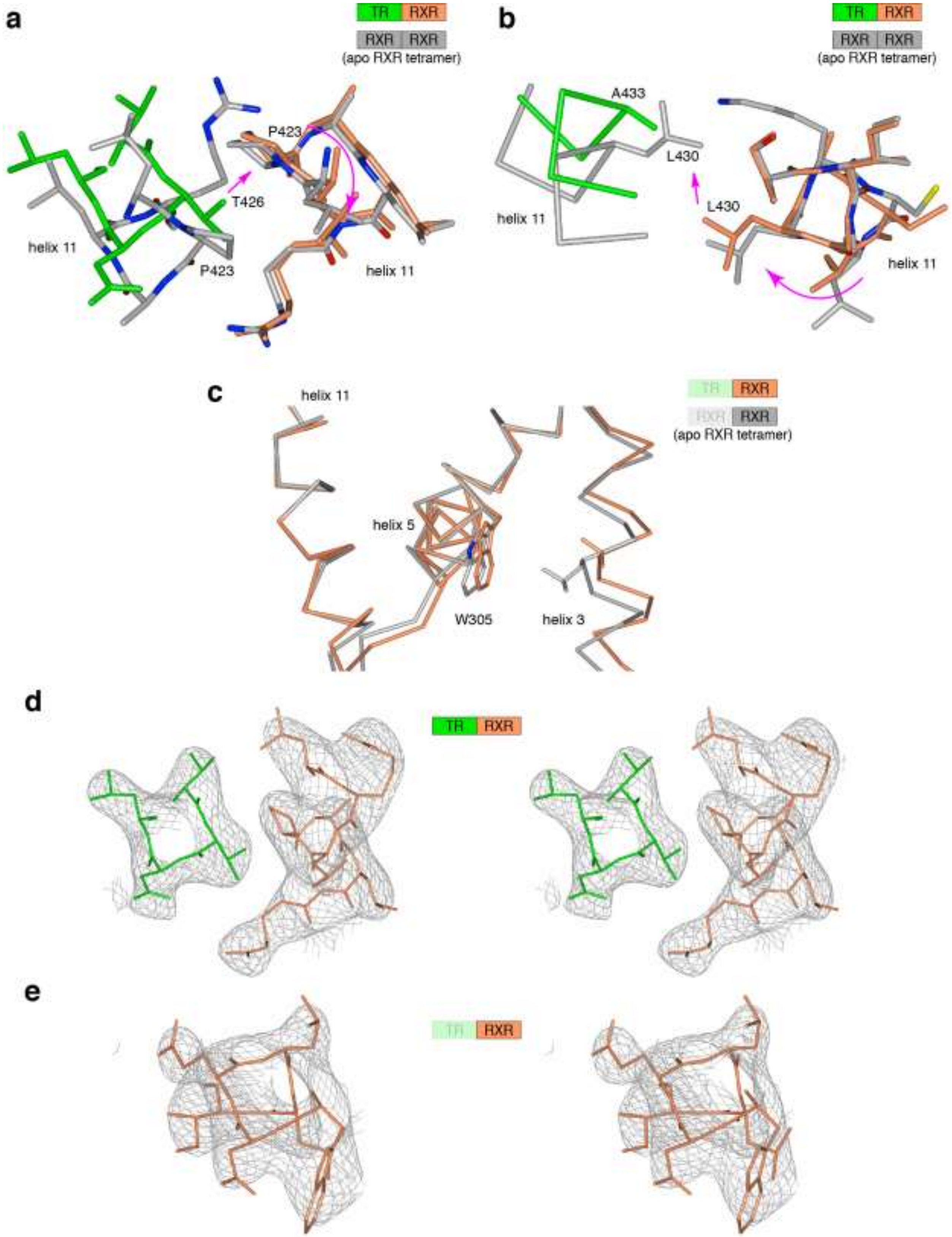


**Supplementary Figure 3. Structural changes in the TR/RXR complex.**

**(a,b)** TR alters RXR conformation via the helix 11 portion of the dimer interface. Apo RXR

homotetramer (PDB 1G1U; colored white) superimposed on TRβ•T3•SRC-2/apo RXRα (colored green and coral, respectively) via RXR. **(a)** The unique position of TR T426 in helix 11 induces a shift in RXR P423 in helix 11 and a rotation of the RXR helical backbone.

**(b)** The repositioning of TR A433 away from the dimer interface, compared to RXR L430, allows RXR L430 and the RXR helical backbone to rotate in the TR/RXR heterodimer.

**(c-e)** Rotation of helix 6 induces an inactive conformation of RXR. **(c)** TRβ•T3•SRC-2/apo RXRα superimposed with apo RXR homotetramer (PDB 1G1U) and drawn as a Cα trace. The rotation of RXR helix 5 in TRβ•T3•SRC-2/apo RXRα puts RXR W305 in position to clash with RXR L276.

**(d,e)** $2F_O\text{-}F_C$ maps for the TRβ•T3•SRC-2/apo RXRα structure are shown at 1σ contour and in cross-eyed stereo for **(d)** the helix 11 dimer interface, centered on RXR P423 with TR colored green and RXR colored coral; and **(e)** RXR helix 5 with W305 in the front.

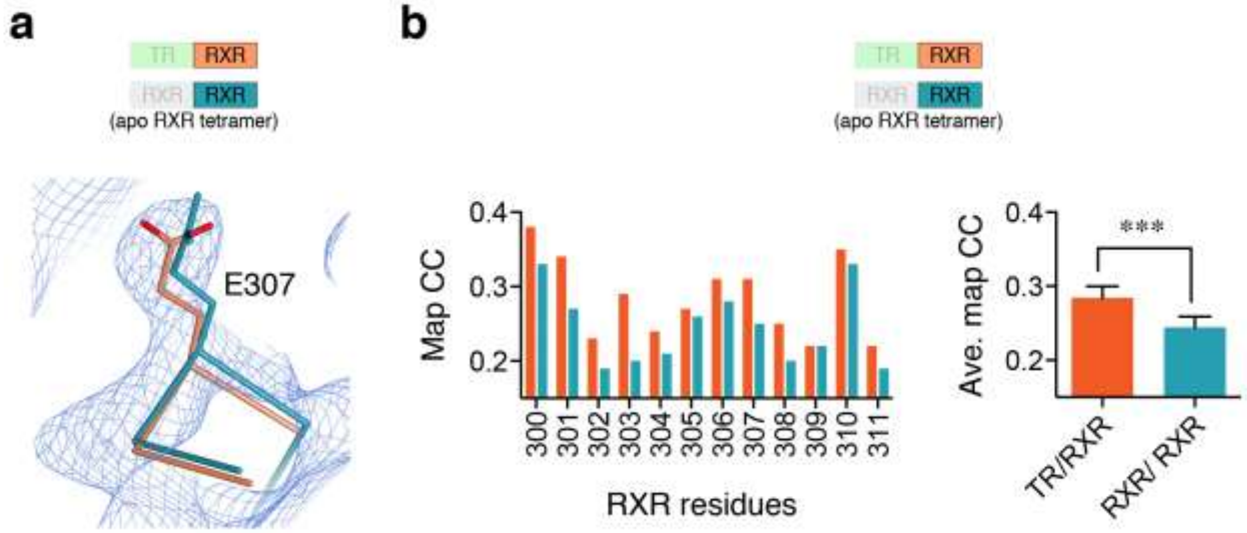


**Supplementary Figure 4. Analysis of the rotation of helix 5 in the TR/RXR structure.**

**(a)** The clear electron density for E307 provides unequivocal support for the positioning of the main chain rotation of helix 5 in this region. The RXR homodimer structure (PDB: 1MVC) was superposed on the new TR/RXR structure (PDB: 4ZO1). The $2F_o$-$F_c$ electron density map of the TR/RXR structure was contoured at $\sigma = 1.0$.

**(b)** Rotation of RXR helix 5 provides a better fit to the electron density map of the TR/RXR structure than a higher resolution RXR structure. Density fit analysis of the superposed structures was performed using *Coot* software. The density fit histogram for RXR helix 5 amino acid residues 300-311 is shown. The average map correlation coefficient (CC) was also calculated (mean ± s.e.m.) for these helix 5 residues. The observed difference in average map CC is statistically significant (paired Students' t-test ***p value = 0.0002).

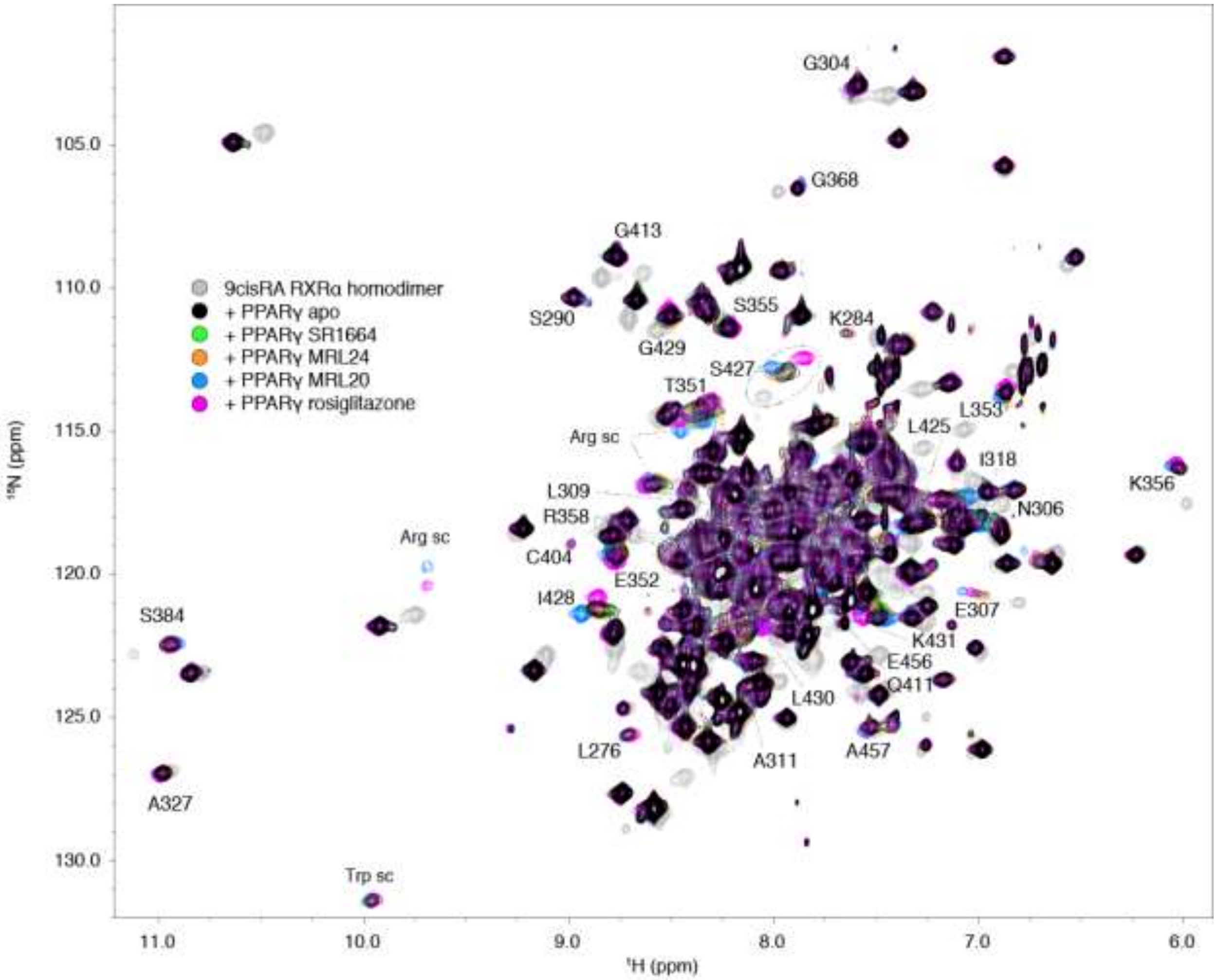


**Supplementary Figure 5. Differential NMR analysis of the effect of the PPARγ ligand binding on the conformation of RXR.**

Overlay of 2D [$^1$H,$^{15}$N]-TROSY-HSQC NMR data for [$^2$H,$^{15}$N]-RXRα LBD in various complexed states, indicated by the legend.

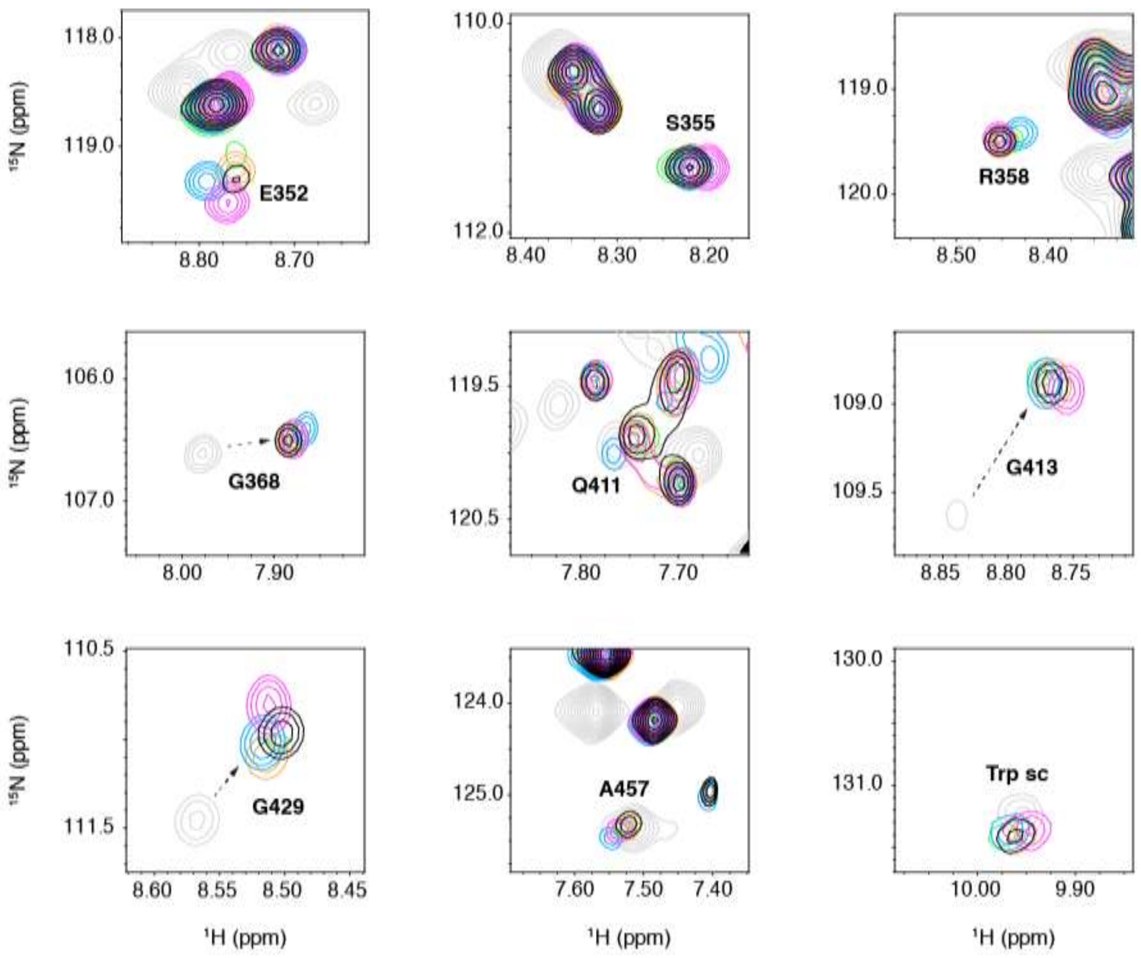


**Supplementary Figure 6. NMR data focusing on other RXR residues affected by ligand binding to PPARγ.**

Same data as in Supplementary Figure 5, but zoomed into regions of interest that are mentioned in the main text.

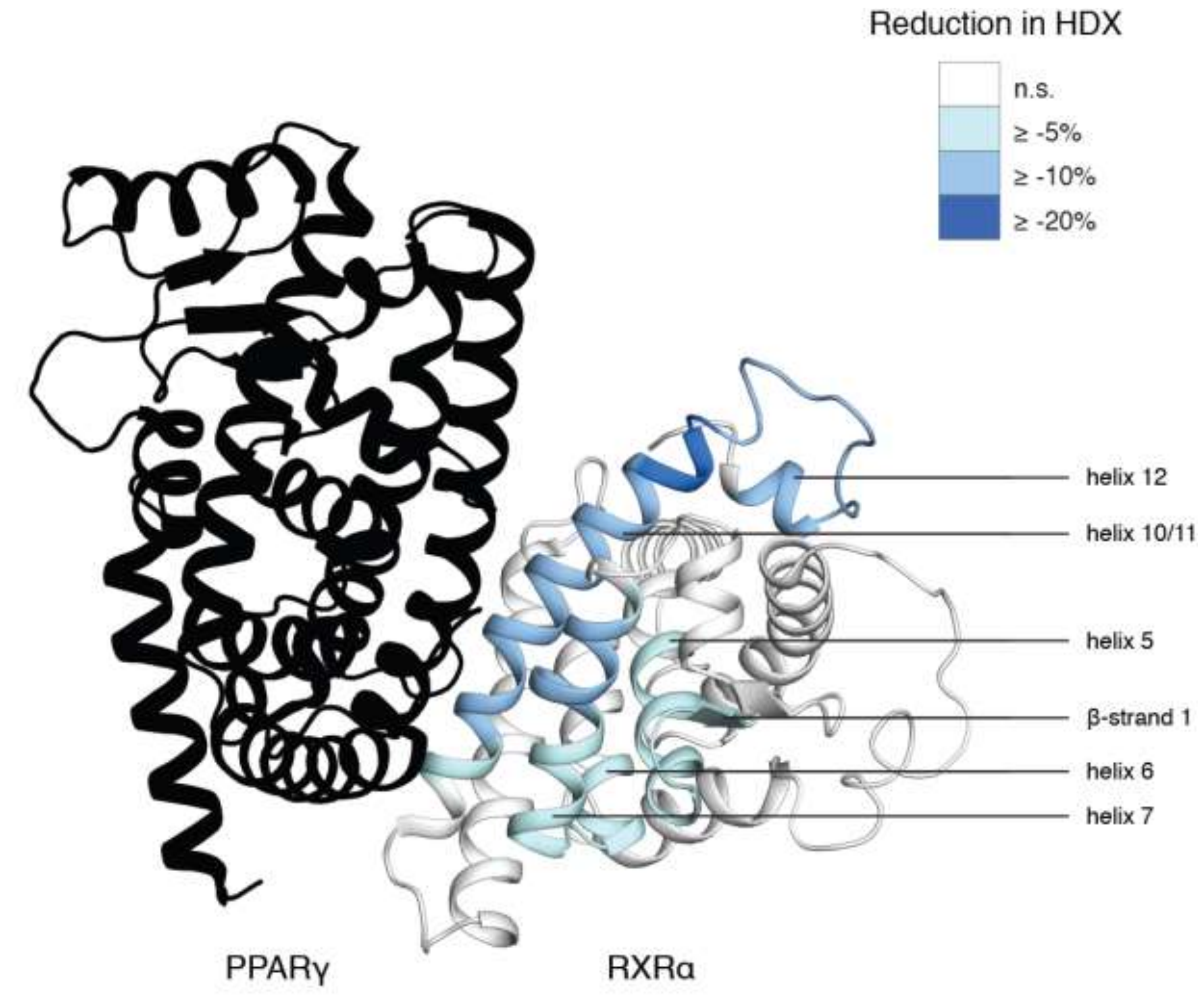


**Supplementary Figure 7. HDX mass spectrometry of the PPARγ/apo-RXRα LBD heterodimer with and without the PPARγ full agonist rosiglitazone.** PPARγ is colored black, RXRα is colored based on percent of HDX in the regions indicated and mapped onto PDB 1FM9 using the color scheme in the legend.

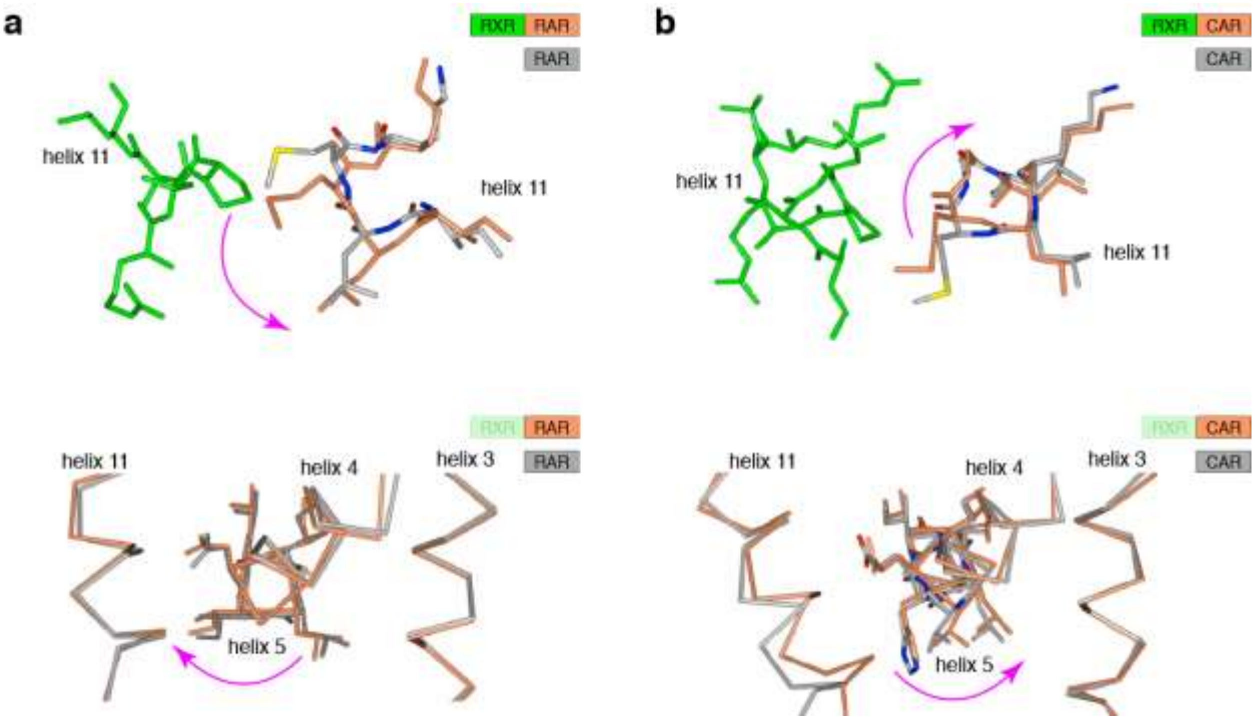


**Supplementary Figure 8. Statistical coupling analysis (SCA) network in other RXR heterodimers**

**(a)** RAR monomer (PDB 1XAP) is colored gray and superimposed on RAR/RXR heterodimer (PDB 1XDK) colored coral and green, respectively. Areas of focus are helix 11 centered at RXR P423 and RAR M372 (top), and the associated rotation of helix 5 (bottom).

**(b)** CAR monomer (PDB 1XNX) is colored and superimposed on CAR/RXR heterodimer (PDB 1XLS) colored coral and green, respectively. Areas of focus are helix 11 centered at RXR P423 and CAR M323 (top), and the associated rotation of helix 5 (bottom). Note that for CAR, the rotations of helix 5 and 11 are in opposite directions from that seen with RAR.

**Supplementary Table 1. HDX values for RXR LBD (±TR LBD)**

| Sequence | z | Start | End | % Change |
|---|---|---|---|---|
| LAVEPKTET | 2 | 240 | 248 | -1 |
| AVEPKTET | 2 | 241 | 248 | 2 |
| AVEPKTETY | 2 | 241 | 249 | 0 |
| AVEPKTETYVEANMGLNPSSPNDPVTNICQ | 3 | 241 | 270 | 0 |
| YVEANMGLNPSSPNDPVT | 2 | 249 | 266 | 0 |
| YVEANMGLNPSSPNDPVTNICQ | 2 | 249 | 270 | 0 |
| YVEANMGLNPSSPNDPVTNICQA | 2 | 249 | 271 | 2 |
| GLNPSSPNDPVTNICQAADKQLFT | 2 | 255 | 278 | 1 |
| AADKQLFT | 1 | 271 | 278 | -4 |
| AADKQLFT | 2 | 271 | 278 | -7 |
| AADKQLFTL | 1 | 271 | 279 | -6 |
| AADKQLFTL | 2 | 271 | 279 | -7 |
| FTLVEWAKRIPHFSEL | 3 | 277 | 292 | -3 |
| LVEWAKRIPHF | 2 | 279 | 289 | 1 |
| LVEWAKRIPHFSEL | 2 | 279 | 292 | -1 |
| LVEWAKRIPHFSEL | 3 | 279 | 292 | -2 |
| LVEWAKRIPHFSELPLDD | 2 | 279 | 296 | -1 |
| LVEWAKRIPHFSELPLDD | 3 | 279 | 296 | -2 |
| LVEWAKRIPHFSELPLDDQ | 2 | 279 | 297 | -1 |
| LVEWAKRIPHFSELPLDDQ | 3 | 279 | 297 | -2 |
| VEWAKRIPHF | 2 | 280 | 289 | -1 |
| VEWAKRIPHFSEL | 2 | 280 | 292 | -2 |
| VEWAKRIPHFSEL | 3 | 280 | 292 | -2 |
| VEWAKRIPHFSELPLDD | 2 | 280 | 296 | 0 |
| VEWAKRIPHFSELPLDD | 3 | 280 | 296 | -1 |
| VEWAKRIPHFSELPLDDQ | 2 | 280 | 297 | -1 |
| VEWAKRIPHFSELPLDDQ | 3 | 280 | 297 | -2 |
| WAKRIPHFSEL | 2 | 282 | 292 | -2 |
| WAKRIPHFSELPLDD | 2 | 282 | 296 | -1 |
| WAKRIPHFSELPLDDQ | 3 | 282 | 297 | -2 |
| SELPLDD | 1 | 290 | 296 | -1 |
| PLDDQ | 1 | 293 | 297 | 1 |
| PLDDQVIL | 1 | 293 | 300 | -3 |
| LRAGWNEL | 1 | 301 | 308 | 0 |
| LRAGWNEL | 2 | 301 | 308 | -2 |
| LRAGWNELL | 2 | 301 | 309 | 0 |
| RAGWNELL | 1 | 302 | 309 | 0 |
| RAGWNELLI | 2 | 302 | 310 | 0 |
| LIASF | 1 | 309 | 313 | -4 |
| LIASFSHRSIA | 2 | 309 | 319 | 1 |
| LIASFSHRSIAVKDGIL | 2 | 309 | 325 | -3 |
| LIASFSHRSIAVKDGIL | 3 | 309 | 325 | -4 |
| LIASFSHRSIAVKDGILL | 3 | 309 | 326 | -5 |

| | | | | |
|---|---|---|---|---|
| IASFSHRSIA | 2 | 310 | 319 | -4 |
| IASFSHRSIAVKDGIL | 3 | 310 | 325 | -5 |
| IASFSHRSIAVKDGILL | 2 | 310 | 326 | -3 |
| ASFSHRSIAVKDGILL | 2 | 311 | 326 | -4 |
| SFSHRSIAVKDGIL | 2 | 312 | 325 | -3 |
| FSHRSIAVKDGIL | 2 | 313 | 325 | -4 |
| SHRSIAVKDGIL | 2 | 314 | 325 | -5 |
| LATGLHVHRNSAHSAGVG | 2 | 326 | 343 | -2 |
| LATGLHVHRNSAHSAGVG | 3 | 326 | 343 | -3 |
| LATGLHVHRNSAHSAGVGAIF | 2 | 326 | 346 | -1 |
| LATGLHVHRNSAHSAGVGAIFD | 3 | 326 | 347 | -2 |
| ATGLHVHRNSAHSAGVG | 2 | 327 | 343 | -1 |
| ATGLHVHRNSAHSAGVG | 3 | 327 | 343 | -3 |
| ATGLHVHRNSAHSAGVGAIF | 3 | 327 | 346 | -2 |
| HVHRNSAHSAGVG | 2 | 331 | 343 | 1 |
| HVHRNSAHSAGVGAIF | 2 | 331 | 346 | 3 |
| DRVLTEL | 1 | 347 | 353 | -8 |
| DRVLTEL | 2 | 347 | 353 | -6 |
| RVLTEL | 1 | 348 | 353 | -6 |
| LVSKMRDMQM | 2 | 353 | 362 | -15 |
| VSKMRDMQM | 2 | 354 | 362 | -15 |
| VSKMRDMQMDKTEL | 2 | 354 | 367 | -7 |
| VSKMRDMQMDKTEL | 3 | 354 | 367 | -8 |
| DKTEL | 1 | 363 | 367 | 0 |
| DKTELGCL | 1 | 363 | 370 | 0 |
| DKTELGCL | 2 | 363 | 370 | -1 |
| FNPDSKGLSNPAE | 2 | 376 | 388 | -2 |
| FNPDSKGLSNPAEVE | 2 | 376 | 390 | -1 |
| FNPDSKGLSNPAEVEA | 2 | 376 | 391 | -3 |
| FNPDSKGLSNPAEVEAL | 2 | 376 | 392 | -4 |
| FNPDSKGLSNPAEVEALREKVYASL | 3 | 376 | 400 | -2 |
| VEALREKVY | 2 | 389 | 397 | -2 |
| VEALREKVYASL | 2 | 389 | 400 | 0 |
| ALREKVY | 2 | 391 | 397 | -2 |
| ALREKVYASL | 2 | 391 | 400 | 0 |
| LREKVYASL | 2 | 392 | 400 | 0 |
| LREKVYASLE | 2 | 392 | 401 | 0 |
| REKVYASL | 1 | 393 | 400 | 0 |
| EAYCKHKYPEQPGRF | 3 | 401 | 415 | -1 |
| AYCKHKYPEQPGRF | 2 | 402 | 415 | -1 |
| YCKHKYPEQPGRF | 2 | 403 | 415 | -2 |
| YCKHKYPEQPGRF | 3 | 403 | 415 | -3 |
| YCKHKYPEQPGRFAKL | 3 | 403 | 418 | -3 |
| CKHKYPEQPGRF | 2 | 404 | 415 | -2 |
| CKHKYPEQPGRF | 3 | 404 | 415 | -2 |
| LLLR | 1 | 418 | 421 | -20 |

| | | | | |
|---|---|---|---|---|
| LLRLPAL | 1 | 419 | 425 | -11 |
| LLRLPAL | 2 | 419 | 425 | -13 |
| LLRLPALRS | 2 | 419 | 427 | -14 |
| LLRLPALRS | 3 | 419 | 427 | -15 |
| LLRLPALRSIGL | 3 | 419 | 430 | -12 |
| LLRLPALRSIGLKC | 2 | 419 | 432 | -13 |
| LLRLPALRSIGLKC | 4 | 419 | 432 | -14 |
| PALRSIGLKC | 3 | 423 | 432 | -19 |
| PALRSIGLKCLEHLFF | 2 | 423 | 438 | -6 |
| RSIGLKCLEHLFF | 2 | 426 | 438 | -5 |
| IGLKCLEHLFF | 2 | 428 | 438 | -5 |
| LEHLFF | 2 | 433 | 438 | 0 |
| FFKLIGDTPIDT | 2 | 438 | 449 | 2 |
| FFKLIGDTPIDTF | 2 | 438 | 450 | -1 |
| FFKLIGDTPIDTFL | 2 | 438 | 451 | 3 |
| FKLIGDTPIDT | 1 | 439 | 449 | 2 |
| FKLIGDTPIDTF | 1 | 439 | 450 | 2 |
| FKLIGDTPIDTFL | 2 | 439 | 451 | 1 |
| IGDTPIDTFL | 1 | 442 | 451 | 0 |
| MEMLEAPHQMT | 2 | 452 | 462 | 1 |
| MLEAPHQMT | 1 | 454 | 462 | 1 |
| MLEAPHQMT | 2 | 454 | 462 | 3 |
| EAPHQMT | 1 | 456 | 462 | 2 |
| APHQMT | 1 | 457 | 462 | 1 |

## Supplementary Table 2. HDX values for PPARγ/RXR LBD (± rosiglitazone)

| Sequence | z | Start | End | % Change |
|---|---|---|---|---|
| RILEAEL | 1 | 234 | 240 | -1 |
| LAVEPKTET | 1 | 240 | 248 | 1 |
| LAVEPKTET | 2 | 240 | 248 | 0 |
| LAVEPKTETY | 2 | 240 | 249 | 0 |
| AVEPKTETYVE | 1 | 241 | 251 | 1 |
| AVEPKTETYVE | 2 | 241 | 251 | 0 |
| AVEPKTETYVEA | 2 | 241 | 252 | 1 |
| YVEANM | 1 | 249 | 254 | -3 |
| YVEANMGLNPSSPNDPVT | 2 | 249 | 266 | 1 |
| YVEANMGLNPSSPNDPVTNICQ | 2 | 249 | 270 | 0 |
| YVEANMGLNPSSPNDPVTNICQ | 3 | 249 | 270 | 0 |
| YVEANMGLNPSSPNDPVTNICQA | 2 | 249 | 271 | 1 |
| YVEANMGLNPSSPNDPVTNICQAADKQLFT | 3 | 249 | 278 | 1 |
| ANMGLNPSSPNDPVTNICQ | 2 | 252 | 270 | 2 |
| GLNPSSPNDPVTNICQ | 2 | 255 | 270 | 1 |
| AADKQLFT | 1 | 271 | 278 | -2 |
| AADKQLFT | 2 | 271 | 278 | -2 |
| AADKQLFTL | 1 | 271 | 279 | -2 |
| AADKQLFTL | 2 | 271 | 279 | -2 |
| LVEWAKRIPHF | 2 | 279 | 289 | -2 |
| LVEWAKRIPHF | 3 | 279 | 289 | -1 |
| LVEWAKRIPHFSEL | 2 | 279 | 292 | -2 |
| LVEWAKRIPHFSEL | 3 | 279 | 292 | -2 |
| LVEWAKRIPHFSELPLDDQ | 2 | 279 | 297 | -1 |
| LVEWAKRIPHFSELPLDDQ | 3 | 279 | 297 | -1 |
| VEWAKRIPHF | 2 | 280 | 289 | -1 |
| VEWAKRIPHF | 3 | 280 | 289 | 0 |
| VEWAKRIPHFSEL | 3 | 280 | 292 | -2 |
| VEWAKRIPHFSELPLDDQ | 2 | 280 | 297 | -1 |
| VEWAKRIPHFSELPLDDQ | 4 | 280 | 297 | -1 |
| LRAGWNEL | 1 | 301 | 308 | 2 |
| LRAGWNEL | 2 | 301 | 308 | 2 |
| LRAGWNELL | 2 | 301 | 309 | 1 |
| RAGWNELL | 1 | 302 | 309 | 2 |
| RAGWNELL | 2 | 302 | 309 | 2 |
| LIASFSHRSIA | 2 | 309 | 319 | -2 |
| LIASFSHRSIA | 3 | 309 | 319 | -5 |
| LIASFSHRSIAVKDGIL | 2 | 309 | 325 | -4 |
| LIASFSHRSIAVKDGIL | 4 | 309 | 325 | -4 |

| | | | | |
|---|---|---|---|---|
| LIASFSHRSIAVKDGILL | 2 | 309 | 326 | -4 |
| LIASFSHRSIAVKDGILL | 3 | 309 | 326 | -5 |
| LIASFSHRSIAVKDGILL | 4 | 309 | 326 | -5 |
| IASFSHRSIA | 1 | 310 | 319 | -5 |
| IASFSHRSIA | 2 | 310 | 319 | -5 |
| IASFSHRSIA | 3 | 310 | 319 | -4 |
| IASFSHRSIAVKDGIL | 2 | 310 | 325 | -4 |
| IASFSHRSIAVKDGIL | 3 | 310 | 325 | -4 |
| IASFSHRSIAVKDGIL | 4 | 310 | 325 | -4 |
| FSHRSIAVKDGIL | 3 | 313 | 325 | -4 |
| SHRSIAVKDGIL | 2 | 314 | 325 | -4 |
| SHRSIAVKDGIL | 3 | 314 | 325 | -4 |
| LATGLHVHRNSAHSAGVG | 3 | 326 | 343 | 0 |
| LATGLHVHRNSAHSAGVGAIF | 3 | 326 | 346 | -1 |
| LATGLHVHRNSAHSAGVGAIF | 4 | 326 | 346 | -1 |
| LATGLHVHRNSAHSAGVGAIF | 5 | 326 | 346 | -1 |
| LATGLHVHRNSAHSAGVGAIFD | 3 | 326 | 347 | -2 |
| LATGLHVHRNSAHSAGVGAIFD | 5 | 326 | 347 | -1 |
| ATGLHVHRNSAHSAGVG | 2 | 327 | 343 | 0 |
| ATGLHVHRNSAHSAGVG | 3 | 327 | 343 | 0 |
| ATGLHVHRNSAHSAGVGAIF | 2 | 327 | 346 | -1 |
| ATGLHVHRNSAHSAGVGAIF | 3 | 327 | 346 | -1 |
| ATGLHVHRNSAHSAGVGAIF | 4 | 327 | 346 | -1 |
| HVHRNSAHSAGVGAI | 3 | 331 | 345 | 1 |
| HVHRNSAHSAGVGAIF | 2 | 331 | 346 | 0 |
| HVHRNSAHSAGVGAIF | 3 | 331 | 346 | -1 |
| FDRVLTEL | 2 | 346 | 353 | -9 |
| DRVLTEL | 1 | 347 | 353 | -10 |
| DRVLTEL | 2 | 347 | 353 | -9 |
| TELVSKMRDMQM | 2 | 351 | 362 | -7 |
| LVSKMRDMQM | 2 | 353 | 362 | -9 |
| VSKMRDMQM | 1 | 354 | 362 | -8 |
| VSKMRDMQM | 2 | 354 | 362 | -8 |
| VSKMRDMQMDKTEL | 2 | 354 | 367 | -5 |
| VSKMRDMQMDKTEL | 3 | 354 | 367 | -4 |
| VSKMRDMQMDKTELGC | 3 | 354 | 369 | -4 |
| VSKMRDMQMDKTELGCLRA | 3 | 354 | 372 | -2 |
| DKTELGCL | 1 | 363 | 370 | 0 |
| DKTELGCL | 2 | 363 | 370 | 0 |
| DKTELGCLRA | 1 | 363 | 372 | 3 |
| FNPDSKGLSNPAEVE | 2 | 376 | 390 | 0 |
| FNPDSKGLSNPAEVEA | 2 | 376 | 391 | -1 |
| FNPDSKGLSNPAEVEAL | 2 | 376 | 392 | 0 |

| | | | | |
|---|---|---|---|---|
| VEALREKVY | 2 | 389 | 397 | 0 |
| VEALREKVYASL | 2 | 389 | 400 | 0 |
| ALREKVY | 1 | 391 | 397 | 0 |
| ALREKVY | 2 | 391 | 397 | 0 |
| ALREKVYASL | 1 | 391 | 400 | 0 |
| ALREKVYASL | 2 | 391 | 400 | 0 |
| ALREKVYASLE | 2 | 391 | 401 | 0 |
| LREKVY | 1 | 392 | 397 | 0 |
| LREKVY | 2 | 392 | 397 | 0 |
| LREKVYASL | 1 | 392 | 400 | 0 |
| LREKVYASL | 2 | 392 | 400 | 0 |
| LREKVYASLE | 2 | 392 | 401 | 0 |
| LREKVYASLEA | 2 | 392 | 402 | 0 |
| REKVYASL | 1 | 393 | 400 | 0 |
| REKVYASL | 2 | 393 | 400 | 0 |
| KVYASL | 1 | 395 | 400 | 0 |
| EAYCKHKYPEQPGRF | 3 | 401 | 415 | -2 |
| EAYCKHKYPEQPGRFAKL | 3 | 401 | 418 | -1 |
| EAYCKHKYPEQPGRFAKL | 4 | 401 | 418 | -1 |
| AYCKHKYPEQPGRF | 3 | 402 | 415 | -2 |
| AYCKHKYPEQPGRFAKL | 4 | 402 | 418 | -1 |
| YCKHKYPEQPGRF | 2 | 403 | 415 | -2 |
| YCKHKYPEQPGRF | 3 | 403 | 415 | -2 |
| YCKHKYPEQPGRF | 4 | 403 | 415 | -2 |
| YCKHKYPEQPGRFAKL | 4 | 403 | 418 | -2 |
| CKHKYPEQPGRF | 2 | 404 | 415 | -2 |
| AKLLLRL | 2 | 416 | 422 | -7 |
| LLRLPAL | 2 | 419 | 425 | -9 |
| LLRLPALRS | 3 | 419 | 427 | -11 |
| LLRLPALRSIG | 3 | 419 | 429 | -10 |
| LLRLPALRSIGL | 2 | 419 | 430 | -12 |
| LLRLPALRSIGLKC | 2 | 419 | 432 | -13 |
| LLRLPALRSIGLKC | 3 | 419 | 432 | -13 |
| LLRLPALRSIGLKC | 4 | 419 | 432 | -12 |
| LLRLPALRSIGLKCLE | 4 | 419 | 434 | -9 |
| PALRSIGLKCLE | 3 | 423 | 434 | -14 |
| PALRSIGLKCLEHLFF | 2 | 423 | 438 | -13 |
| PALRSIGLKCLEHLFF | 4 | 423 | 438 | -13 |
| RSIGLKC | 2 | 426 | 432 | -16 |
| RSIGLKCLEHLFF | 2 | 426 | 438 | -15 |
| IGLKCLEHLFF | 3 | 428 | 438 | -12 |
| LKCLEHLFF | 2 | 430 | 438 | -11 |
| LEHLF | 2 | 433 | 437 | -11 |

| | | | | |
|---|---|---|---|---|
| LEHLFF | 2 | 433 | 438 | -14 |
| HLFF | 1 | 435 | 438 | -20 |
| FFKLIGDTPIDTF | 2 | 438 | 450 | -10 |
| FFKLIGDTPIDTFL | 2 | 438 | 451 | -8 |
| FKLIGDTPID | 1 | 439 | 448 | -9 |
| FKLIGDTPID | 2 | 439 | 448 | -8 |
| FKLIGDTPIDT | 1 | 439 | 449 | -7 |
| FKLIGDTPIDT | 2 | 439 | 449 | -7 |
| FKLIGDTPIDTF | 1 | 439 | 450 | -7 |
| FKLIGDTPIDTF | 2 | 439 | 450 | -7 |
| FKLIGDTPIDTFL | 1 | 439 | 451 | -9 |
| FKLIGDTPIDTFL | 2 | 439 | 451 | -16 |
| FLMEM | 1 | 450 | 454 | -13 |
| MEMLEAPHQMT | 2 | 452 | 462 | -1 |
| MLEAPHQMT | 1 | 454 | 462 | -1 |
| MLEAPHQMT | 2 | 454 | 462 | -1 |
| LEAPHQMT | 1 | 455 | 462 | -1 |
| LEAPHQMT | 2 | 455 | 462 | -1 |
